# Development of a hookworm egg hatching assay to determine the ovicidal effects of anthelminthics

**DOI:** 10.1186/s13071-023-05771-8

**Published:** 2023-05-04

**Authors:** Erin Easland, Stefan Biendl, Jennifer Keiser

**Affiliations:** 1 Department of Medical Parasitology and Infection Biology, Swiss Tropical and Public Health Institute, Kreuzstrasse 2, 4123 Allschwil, Switzerland; 2 University of Basel, 4003 Basel, Switzerland

**Keywords:** Anthelminthics, Drug discovery, Hookworm, Egg hatching, *Heligmosomoides polygyrus*, *Necator americanus*, *Ancylostoma duodenale*

## Abstract

**Background:**

Few anthelminthics are currently available, manifesting the urgent need for new treatment options. In vitro profiling of current anthelminthics against larval and adult stage helminths displayed varying effects on closely related worm species and between life stages of the same species. Conversely, limited research has been performed on the egg stage of human hookworms, and the effects of investigational compounds on the egg stage are not routinely assessed.

**Methods:**

We profiled the development and hatching of *Heligmosomoides polygyrus*, *Ancylostoma duodenale* and *Necator americanus* eggs isolated from rodent faeces in liquid media with various nutrient levels, osmolar concentrations, and acidities in dependence on incubation temperature and light exposure. Incubation conditions were optimised to allow the study of drug effect on immature and embryonated eggs. We analysed concentration-effect relationships of commercially available anthelminthics over 72 h.

**Results:**

Rapid embryonation and hatching were observed at room temperature with and without light exposure without nutrient supplementation in a wide range of acidities. Hookworms hatched optimally at room temperature in PBS achieving > 75% hatching over 34 h. Developmental delays were seen when eggs were stored at 4 °C with no effect on viability. Similar delays were also seen with increased osmolar concentrations resulting in decreased viability. Benzimidazole anthelminthics effectively reduced the viability and prevented hatching of hookworm eggs, with albendazole and thiabendazole eliciting particularly potent effects at EC_50_ values below 1 µM. Macrolide anthelminthics as well as emodepside, oxantel pamoate, and pyrantel pamoate were inactive while monepantel, levamisole, and tribendimidine displayed varied potencies among the hookworm species.

**Conclusion:**

The presented egg-hatching assay will complement ongoing anthelminthic drug discovery and allow a full characterisation of drug activity against all life stages. In the development and application of the egg-hatching assay, good accordance was observed between the three hookworm species evaluated. Marketed anthelminthics show differences of drug action compared to larval and adult stages highlighting the importance of profiling drug activity against all life stages.

**Graphical Abstract:**

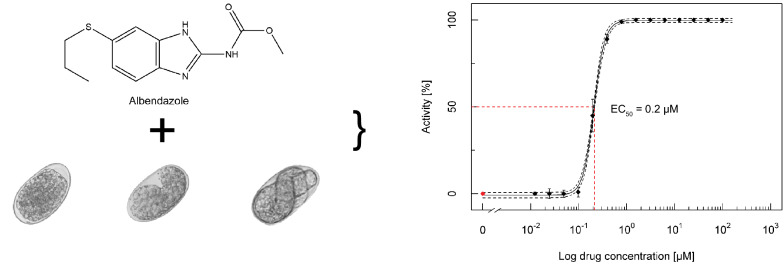

**Supplementary Information:**

The online version contains supplementary material available at 10.1186/s13071-023-05771-8.

## Background

The human hookworms *Necator americanus* and *Ancylostoma duodenale* infect about 470 million people worldwide and are primarily endemic in poverty-stricken areas of tropical and sub-tropical climates [1, 2]. Infection initiated by skin penetration leads to invasion of the lungs and intestine, resulting in painful lung and intestinal manifestations and anemia [1–3]. Within the host, adult hookworms release thousands of eggs daily, which exit through faeces and continue the life cycle and cause subsequent infections [1]. Currently, mass drug administration (MDA) is fundamental to control infection and reduce morbidity [1, 4]; however, only few chemotherapeutic agents are currently available against these helminths [5, 6]. The standard therapy used for treatment includes drugs derived from anthelminthics for veterinary use, primarily albendazole and mebendazole of the benzimidazole family [5]. Monotherapies administered at single doses display only moderate cure rates and efficacy varies over studied regions [7]. Additionally, pharmacotherapy does not prevent reinfection [8] and no licensed vaccines are currently available [9, 10]. Since there is an urgent need for new treatment options, and anthelminthic drug discovery is underfunded and neglected [4, 6, 11], it is essential to optimise drug discovery.

Current methods in helminth drug discovery overview a variety of phenotypic [12–14] and motility [14–18] based assays that have been developed and advanced to detect nematocidal activity primarily using larval and adult stages [10]. However, only few studies published incorporate the use of egg hatching to determine ovicidal abilities in drug discovery, omitting a part of the life cycle [10, 19]. Nevertheless, similar methodologies have been of use for over 40 years in the detection of helminth strains resistant to treatment [20–22]. The establishment of an egg-hatching assay platform for human hookworms harbors the potential to detect cryptic ovicidal effects of extant compounds, complementing anthelminthic drug screening.

The aim of our study was to enable the complete characterisation of anthelminthics against all life stages of hookworms by targeting the understudied egg stage. We developed an in vitro assay for drug evaluation against the egg stage through optimisation of laboratory conditions of hookworm egg maturation and hatching of *Heligosomoides polygyrus*, *Ancylostoma ceylanicum*, and *N. americanus*. We evaluated both unembryonated and embryonated eggs to consider drug sensitivity differences based on egg maturity. The anthelminthics assessed in this study included key benzimidazoles and macrolides and further commercially available anthelminthics, namely monepantel, levamisole, tribendimidine, emodepside, oxantel pamoate, and pyrantel pamoate. Full characterisation of the potency of broadly employed anthelminthics will help answer why drug action differs over life stages and in translation between host species as well as test whether this assay could be integrated in hookworm drug discovery [23].

## Methods

### Animals

Female mice (NMRI strain; age 3 weeks; weight ca. 20 − 22 g) were purchased from Charles River (Sulzfeld, Germany) and male Syrian Golden hamsters were purchased from Janvier Laboratories (Le Genest-Saint-Isle, France). Rodents were kept in polycarbonate cages under environmentally controlled conditions (temperature ∼25 °C; humidity ∼70%; 12 h light:12 h dark cycle) with free access to water and food. The rodents were acclimatised for 1 week before infection.

## Compounds and culture media

Abamectin, albendazole, doramectin, eprinomectin, fenbendazole, flubendazole, ivermectin, levamisole hydrochloride, mebendazole, oxfendazole, oxibendazole, pyrantel pamoate, ricobendazole, and thiabendazole were the products of Sigma-Aldrich (Buchs, Switzerland). Milbemycin oxime and moxidectin were purchased from Hangzhou Dingyan Chemical Co., Ltd. (Hangzhou, China). Tribendimidine was obtained from Shandong Xinhua Pharmaceutical Co., Ltd. (Zibo, China).

The compounds were dissolved in pure DMSO (Sigma-Aldrich, Switzerland) at a concentration of 10 mM, aliquoted and stored at − 20 °C until further use.

Phosphate-buffered saline (PBS, Sigma-Aldrich) used for conditional testing of egg hatching as well as the in vitro egg-hatch drug assays was prepared by supplementing PBS with 1% penicillin (10,000 U/ml) and streptomycin (10 mg/ml) solution (Sigma-Aldrich) and 5% amphotericin B (250 μg/ml, Sigma-Aldrich).

Hanksʼ balanced salt solution (HBSS; Gibco, Waltham MA, USA) and RPMI 1640 culture medium (Gibco) were additionally used for conditional testing of egg hatching and were prepared by supplementing with 1% penicillin (10,000 U/ml) and streptomycin (10 mg/ml) solution (Sigma-Aldrich) and 5% amphotericin B (250 μg/ml, Sigma-Aldrich).

All media were sterilised by filtration using a 0.22-μm filter bottle (Vitaris AG, Baar, Switzerland).

## Parasites

The life cycles of the hookworms *H. polygyrus*, *N. americanus*, and *A. ceylanicum* are established and maintained at Swiss TPH (Keiser et al. 2021). *Heligosomoides polygyrus* infective stage larvae (L3) (*n* = 100) were orally given to female NMRI mice and *N. americanus* (*n* = 150) and *A. ceylanicum* L3 (*n* = 140) were subcutaneously and orally administered, respectively, to male Syrian golden hamsters. Starting at 2–6 weeks post-infection depending on the species, hookworm eggs used for in vitro assays were collected by filtering the faeces of infected hamsters (*A. ceylanicum* and *N. americanus*) and mice (*H. polygyrus*). Further purification was performed using floatation in a saturated sodium nitrate suspension, followed by two washing cycles in PBS. Purified eggs were counted and diluted to a suspension of 0.7 eggs/µl in supplemented PBS.

For in vitro assays with unembryonated eggs of *H. polygyrus*, *N. americanus*, and *A. ceylanicum*, eggs were used immediately following isolation and purification from faeces. For in vitro assays with embryonated eggs of *H. polygyrus*, the unembryonated eggs obtained after isolation and purification were left to mature at 4 °C for 5–6 days and utilised in assays subsequently.

## Characterisation and optimisation of determinants of hookworm egg hatching

Optimisation of in vitro egg hatching and establishment of optimal conditions for an in vitro egg-hatch assay were conducted by phenotypic characterisation of egg development of unembryonated or embryonated *H. polygyrus* eggs and quantifying hatching rates in several conditions.

To assess egg hatching in a spectrum of nutrient rich and minimal liquid media and a range of temperature, 30–40 eggs were suspended in 200 µl of PBS, HBSS, or RPMI within sterile 96-well flat bottom plates, incubated at 4 °C, 21 °C (room temperature), and 37 °C and the number and morphology of eggs hatched were quantified daily for 12 days under an inverted transmitted-light microscope at 10 × magnification. For eggs exposed to 37 °C, the viability of unhatched eggs was determined after an additional period of 24 h at room temperature.

To study light sensitivity of *H. polygyrus* embryonic development and hatching, eggs were incubated in PBS at room temperature with exposure to a constant light source or protected from light. The numbers of hatched, embryonated, and unembryonated eggs were assessed every 2 h until no further hatching occurred.

To study *H. polygyrus egg* maturation and hatching at 4 °C, eggs were suspended in cold PBS directly after isolation, and incubated at 4 °C. *Heligmosomoides polygyrus* eggs were assessed by evaluating morphology of eggs at each stage of embryonation over 6 days and quantifying the number of eggs hatched daily over 2 weeks.

For assessing the influence of media osmolality, viability of eggs and their hatching percentage rate were evaluated in purified water with increasing NaCl supplementation (0%, 0.5%, 0.9%, 1.8%, 2.5%, 5%, 10%, and 25%) at room temperature. For assessing the influence of pH on viability, and hatching percentage, eggs were assessed in PBS adjusted to a range of acidities (pH 2, 4, 7.4, 9) at room temperature. The acidity was adjusted using HCl (7.78 M) and NaOH (0.1 M). After 24 h, the viability of unhatched eggs among tested conditions was determined by resuspending eggs in purified water and quantifying additional hatching after 24 h.

Additionally, maturation and hatching rates of *N. americanus* and *A. ceylanicum* eggs were assessed in PBS, HBSS, and RPMI prior to conducting assays with compounds. Furthermore, optimal assay conditions determined with *H. polygyrus* eggs were assessed with *N. americanus* and *A. ceylanicum* when adapting the in vitro egg-hatch drug assays.

Conditions were assessed in triplicate and repeated at least once with eggs of each hookworm species.

## In vitro phenotypic drug sensitivity assays

A range of anthelminthic compounds and derivatives were screened against unembryonated eggs of *H. polygyrus*, *N. americanus*, and *A. ceylanicum* as well as embryonated eggs of *H. polygyrus*. To determine EC_50_ values, 30 to 40 hookworm eggs were incubated in drug concentrations obtained from two-fold serial dilutions for a total of at least eight concentrations starting at 100 µM (Additional file 1: Table S1). For each prepared assay, the highest concentration of DMSO in culture medium served as negative control. Assay plates were incubated at room temperature, protected from light, and evaluated at 72 h. The total number of eggs and those that hatched were quantified under an inverted transmitted-light microscope at 10 × magnification (Carl Zeiss, Germany, magnification 10–40 ×). Each experimental condition was tested in triplicate and experiments were repeated at least once.

## Data analysis

Egg development and hatching percentage were determined by counting the number of unembryonated, embryonated, and hatched eggs compared to the total number of eggs. Drug activity was determined by normalising hatching counts of the eggs exposed to drugs to hatching counts of the vehicle-control and averaging across replicates.

The EC_50_ values of the tested drugs were determined by applying a nonlinear least-squares analysis using a four-parameter sigmoid function. Drug activities and EC_50_ values were calculated in R (version 4.0.3) as previously described [24].

Visualisations were created in R and GraphPad Prism (version 8.2.1).

## Results and discussion

### Characterisation and optimisation of determinants of hookworm egg hatching

Efficient and reproducible hookworm egg hatching in liquid medium is an important prerequisite for the development of an in vitro egg hatching assay. Current standard practice of hatching hookworm eggs in controlled laboratory conditions [25] to obtain larvae (for rodent infections or larval based assays) is unsuitable for use in a drug activity assay as non-purified egg suspensions are poured over Petri dishes to hatch and mature over 1 week. Therefore, we first determined the ability of hookworm eggs to effectively hatch within liquid media, specifically PBS, HBSS, and RPMI, selected for assessment because of their use for incubation of hookworm larvae. The percent of eggs that hatched was observed to be equally high (> 75%) among each medium tested, independent of media nutrient richness (Additional file 1: Fig S2). We selected PBS for further experiments to minimise media influence on compound effect while maintaining a constant pH to reduce influence from unspecific acidic/basic properties of test compounds.

**Fig. 1:**
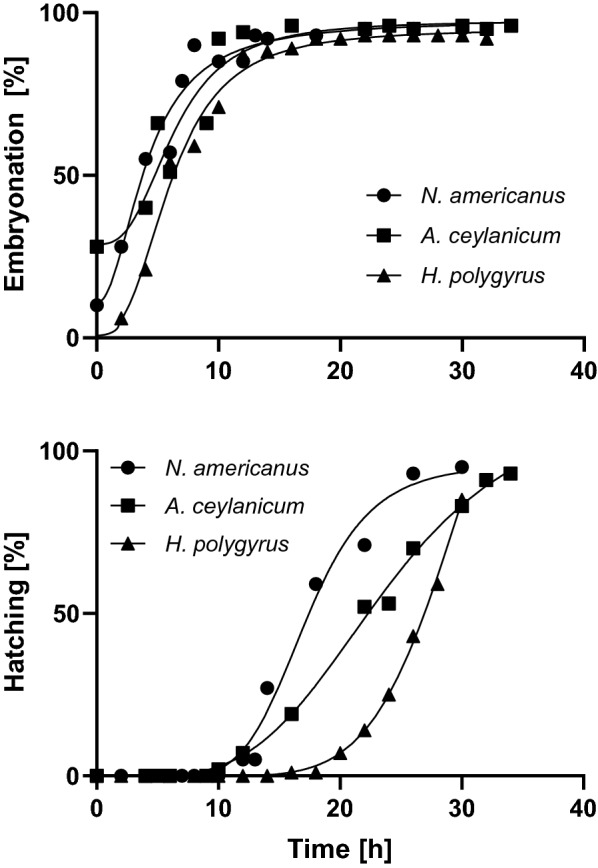
In vitro maturation (top panel) and hatching (bottom panel) of *N. americanus*, *A. ceylanicum,* and *H. polygyrus* eggs at room temperature. Each point represents the mean percentage of embryonated or hatched eggs and lines represent the best fit

To establish assay read-out time points as well as to observe changes in development, we studied hookworm maturation and hatching over time in assay media at room temperature (Fig. 1). Comparable maturation and hatching rates were observed among the three hookworm species tested. Directly following isolation, *N. americanus* and *A. ceylanicum* eggs were presenting moderate levels of embryonic development (28–60%), differing from *H. polygyrus* eggs for which first stages of embryonic development were observed from 4 h after isolation. Complete embryonation of eggs of each species was observed at 12 h, also commencing the beginning of hatching (stages pictured in Additional file 1: Fig S1). Faster initial hatching rates occurred among *N. americanus* and *A. ceylanicum* compared to *H. polygyrus*, likely due to the higher levels of embryonation at zero hours; however, completion of hatching occurred among all species between 30 and 34 h.

To determine whether light exposure stimulates hatching as seen with other helminth species [11], *H. polygyrus* eggs were evaluated with and without light exposure. Light exposure did not influence the hatching, independent of maturation status of eggs (Additional file 1: Fig S3). Nevertheless, subsequent assays were performed protected from light for stringent control of environmental conditions between assays.

We further assessed incubation of freshly isolated eggs at 4 °C, aiming at preventing the rapid maturation and hatching of *H. polygyrus* eggs without compromising their viability [11, 26], thus allowing for increased temporal flexibility of drug assay execution. Early stages of embryonation were exhibited within 6 h at 4 °C compared to 4 h at room temperature, and complete maturation of viable eggs occurred over 5 days longer than seen at room temperature (Additional file 1: Fig. S4). The hatching process of the eggs was delayed within the cold temperatures accordingly. The eggs at room temperature hatched over 1–2 days and those at 4 °C hatched over 1–2 weeks (Additional file 1: Fig S5). Incubation of the eggs at 4 °C over 2 weeks did not negatively affect the eggs, presenting an abundance of eggs still intact and viable after 9 days. Over half the eggs had hatched by 10 days and maturation of the remaining eggs was well advanced. No loss of viability was detected at 4 °C as we observed complete hatching (95 ± 2%) of incubated eggs at 4 °C within 2 weeks. Additionally, eggs fully matured at 4 °C and brought back to room temperature displayed hatching rates of > 95%, similar to those embryonated at room temperature (Additional file 1: Fig S3). Overall, storage at 4 °C allowed delaying the maturation and hatching of *H. polygyrus* eggs, enabling flexible assay set up and experimentation of eggs at varying maturities. Similar assessment of 4 °C incubation with *N. americanus* and *A. ceylanicum* eggs displayed lack of viability, resulting in the need to set up assays directly after egg isolations (data not shown). Their increased sensitivity to lower temperatures compared to *H. polygyrus* eggs is not understood, but is consistent with temperature sensitivity discrepancies of the larvae among these three species.

Incubating *H. polygyrus* eggs at 37 °C induced deterioration of embryo morphology after 24 h and prevented hatching, even after returning eggs to room temperature (data not shown).

We further assayed alternative conditions to delay hatching of embryonated *H. polygyrus* eggs, such as varying hatching media osmolarity and acidity. As increased medium osmolarity has been previously described to delay the quick hatching of *Schistosoma* eggs [11, 27], we assessed the effect of medium osmolarity on *H. polygyrus* egg hatching. In hypo-osmolar conditions the eggs hatched as observed within iso-osmolar concentrations (Additional file 1: Figure S6). In hyper-osmolar conditions, however, hatching delays coupled to decreased egg viability were observed and this effect increased with increases in media salinity (Additional file 1: Fig S7 and S8). The reduction in viability of eggs in hyper-osmolar environment was also visualised with morphological differences among the unhatched eggs (Additional file 1: Fig S8). Due to the diminishing effect on *H. polygyrus* eggs, media with increased osmolar concentration were not further used for storing the hookworm eggs.

In contrast to their vulnerability to changes in media osmolarity, *H. polygyrus* eggs maintained high viability over a wide pH range. Examining the effect of variation in media pH, we observed no delay in hatching or change in viability at a pH range of four to nine (Additional file 1: Fig S7). Our findings are consistent with previous studies showing that hookworms can tolerate pH ranges between 4.6 and 9.4 [28] and relate to naturally occurring variability in soil acidity, ranging from a pH of 3.5 to 10 [29]. Overall, slightly acidic soil near pH 6 has been identified to be optimal for egg hatching in areas of high hookworm prevalence [28]. At pH 2, we observed deterioration of the embryo (Additional file 1: Fig S8) with consequently no hatching and no restoration of viability after neutralisation of the media. 

Based on the determinants discovered in experimentation of hookworm egg maturation and hatching, assay set-up and time points were selected to develop a robust and precise in vitro egg-hatching assay. The assays were conducted over 72 h to quantify all possible viability changes of the eggs as in this period of time most eggs had hatched among the selected conditions at room temperature. In implementing a straightforward, unbiased scoring method, data retrieved from assays also display high specificity. The accuracy of the assays could not be defined as the activity on the egg stage was previously unknown, but the range of obtained results showed 0% to 100% activity among tested compounds as well as expected lack of activity among the controls.

## Anthelminthic compound profiling on hookworm eggs

The developed egg-hatching assay was utilised to broaden the scope of known anthelminthic activity to hookworm egg development and hatching by evaluating the activities of commercially available anthelminthics (Additional file 1: Fig S9) on the egg stage. EC_50_ values of compounds assessed against the eggs of *N. americanus*, *A. ceylanicum*, and *H. polygyrus* are summarised in Table 1. In general, individual drug action was equivalent among the three tested species, further confirming *H. polygyrus* as an excellent model organism for drug discovery against hookworms.

**Table 1:** In vitro activity (EC_50_ values [µM]) of anthelminthics against eggs of *Necator americanus, Ancylostoma ceylanicum*, and *Heligmosomoides polygyrus*, determined after 72 h of drug exposure

Drug family	Compound	*N. americanus* (unembryonated)	*A. ceylanicum* (unembryonated)		*H. polygyrus* (unembryonated)	*H. polygyrus* (embryonated)
		EC_50_ [µM] (s.e.)	EC_50_ [µM] (s.e.)		EC_50_ [µM] (s.e.)	EC_50_ [µM] (s.e.)
Benzimidazoles	Thiabendazole	0.27 (0.02)	0.27 (0.01)		0.21 (0.01)	0.41 (0.03)
	Albendazole	0.48 (0.03)	0.40 (0.03)		0.42 (0.01)	0.49 (0.03)
	Oxibendazole	2.6 (0.3)	1.53 (0.11)		0.70 (0.01)	1.43 (0.08)
	Mebendazole	4.9 (0.3)	1.15 (0.13)		4.6 (0.1)	8.4 (1.1)
	Flubendazole	3.6 (3.5)	32.9 (3.3)		4.9 (0.3)	8.9 (0.6)
	Albendazole sulfoxide	> 100	> 100		> 100	> 100
	Fenbendazole	> 100	> 100		> 100	> 100
	Oxfendazole	> 100	> 100		> 100	> 100
Macrolides	Abamectin	> 100	> 100		> 100	> 100
	Doramectin	> 100	> 100		> 100	> 100
	Eprinomectin	> 100	> 100		> 100	> 100
	Ivermectin	> 100	> 100		> 100	> 100
	Milbemycin oxime	> 100	> 100		> 100	> 100
	Moxidectin	> 100	> 100		> 100	> 100
Miscellaneous	Monepantel	51.4 (33.8)	1.05 (0.09)		> 100	1.86 (0.42)
	Levamisole	12.6 (1.9)	5.6 (0.5)		14.9 (1.0)	5.1 (0.5)
	Tribendimidine	44.4 (5.7)	> 100		85.3 (4.8)	48.4 (12.5)
	Emodepside	> 100	> 100		> 100	> 100
	Oxantel pamoate	> 100	> 100		> 100	> 100
	Pyrantel pamoate	> 100	> 100		> 100	> 100

(Standard error (s.e.) of the mean in bracket)

**Fig. 2:**
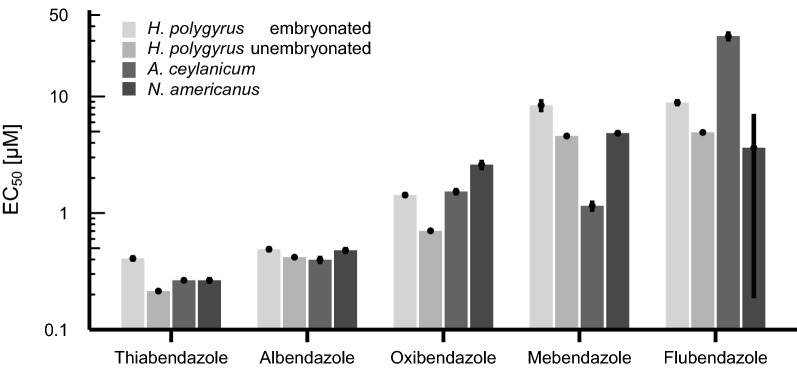
Benzimidazole activity against unembryonated *N. americanus*, *A. ceylanicum*, and *H. polygyrus* eggs and embryonated *H. polygyrus* eggs after 72 h of drug exposure. Each point represents the mean EC_50_ value [µM] with error bars representing the standard error (s.e.) of the mean

**Fig. 3:**
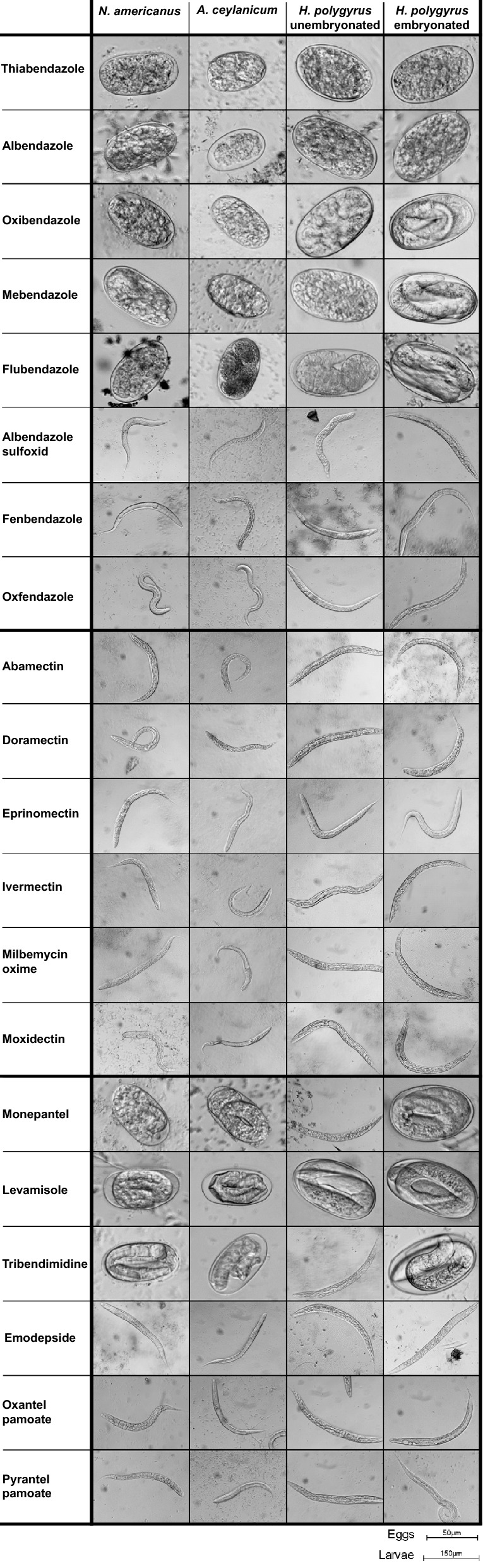
Effect of benzimidazoles, macrolides, and miscellaneous anthelminthics on unembryonated *N. americanus*, *A. ceylanicum*, and *H. polygyrus* and embryonated *H. polygyrus* eggs after 72 h of drug exposure at 100 µM. Eggs are depicted for active drugs preventing hatching, while larvae are depicted for inactive drugs

The benzimidazoles displayed the highest potency among the hookworm eggs tested against (Fig. 2), with thiabendazole and albendazole in particular featuring EC_50_ values < 1 µM against all hookworm eggs. Deterioration of the hookworm embryos was observed among the active benzimidazoles with the severity of deterioration correlating to the level of potency quantified (Fig. 3). Only three of the eight benzimidazole compounds were not active, including albendazole sulfoxide, fenbendazole, and oxfendazole, displaying primarily the hatching of normal appearing larvae (Fig. 3). A comparable lack of activity of these oxidised metabolites was observed in a similar study assessing activity against *Strongyloides ratti* adults within PBS media [23], suggesting a potential phosphate-related quenching of drug action. Importantly, active benzimidazoles were more potent against unembryonated than against embryonated eggs of *H. polygyrus*. Additionally, in contrast to their activity on the egg stage, the benzimidazoles show generally low potency against larval and adult stages of hookworm [23]. Tubulin synthesis is especially important during development and maturation of the embryo and the benzimidazoles act to inhibit microtubule synthesis, hence destroying the cell structure and causing death of the parasites [30]. Since the eggshell appears to be unable to protect the developing larvae within, the egg stage is particularly sensitive to benzimidazole exposure.

All six macrolides tested displayed no activity against the hookworm eggs (all EC_50_ values > 100 µM). Complete hatching was observed among these compounds after 72 h (Fig. 3). Macrolides feature much larger structures than the benzimidazoles (Additional file 1: Fig S9b), revealing a possible steric cause for the lack of ovicidal activity. In contrast to the benzimidazoles the protective barrier of the shell may be preventing entry of the drug and exposure of the developing larvae within. Additionally and in contrast to hookworm larvae susceptible to macrolides [23, 30], the developing larvae within the egg likely do not depend on the feeding and motility facilitated by the macrolide target glutamate and GABA channels [30].

Among the miscellaneous anthelminthic compounds tested, monepantel, levamisole, and tribendimidine displayed varied potencies among the hookworm species. Monepantel has been previously described with activity only against *A. ceylanicum* L3 [23], correlating with the highest potency observed among *A. ceylanicum* eggs. High potency was also observed against embryonated *H. polygyrus* eggs, although no activity was displayed against the unembryonated eggs. Lower potency of monepantel was also observed against *N. americanus* eggs, not seen previously against its L3 or adult stages [23]. Levamisole and tribendimidine act on the same receptor [31] presenting overall high potencies among hookworm L3 and adult parasites [23]. This activity was also seen among the hookworm eggs with overall lower potency of tribendimidine compared to levamisole. These three active compounds did not display the level of embryonic deterioration observed among the active benzimidazoles as many remaining unhatched eggs exhibited clear embryonation (Fig. 3). Emodepside, oxantel pamoate, and pyrantel pamoate displayed no activity against the hookworm eggs (all EC_50_ values > 100 µM) with complete hatching observed by 72 h (Fig. 3). Further research is needed to describe the lack of activity seen with these compounds which may be of interest in explaining the difference in activity seen compared to compounds such as levamisole and tribendimidine found active with similar mechanisms of action [32].

## Conclusion

In summary, we developed and utilised egg-hatching assays to broaden the understanding of anthelminthic drug activity on hookworm egg maturation and hatching. Hookworms hatched optimally at room temperature in PBS achieving > 75% hatching over 34 h. Storing eggs at 4 °C proved beneficial to study drug effect on embryonated eggs by delaying maturation and hatching while preserving egg viability with > 95% hatching observed within 2 weeks or when returned to room temperature.

The in vitro assessment of the hookworm eggs showed further differences of drug action compared to larval and adult stages, displaying a changed sensitivity to the tested compounds within the eggshell. The benzimidazoles displayed the strongest potency against the egg stage, in particular thiabendazole and albendazole with EC_50_ values < 1 µM. Conversely, the macrolides displayed no activity although previously activity was seen against adults and particular high potency against larvae (23). Emodepside, oxantel pamoate, and pyrantel pamoate were inactive while monepantel, levamisole, and tribendimidine displayed varied potencies among the hookworm species. The activity discrepancies of currently profiled anthelminthics observed between life stages highlight the importance of profiling drug activity against all life stages, including the egg-stage model, in drug discovery for human hookworms. Likewise, we would not recommend screening compounds against the egg stage solely, even though this might be the most cost-effective method. Also, in adapting the developed assay from *H. polygyrus* to *N. americanus* and *A. ceylanicum*, assay conditions and determinants did not need to be altered, displaying good accordance among the three parasite models. Additionally, the resulting data from the assays were predominantly similar among the three species, further verifying *H. polygyrus* and *A. ceylanicum* as excellent models in hookworm drug discovery.

Continuing application of this egg-hatching assay will aid in enabling further drug discovery by identifying ovicidal abilities of known anthelminthics and potential drug candidates.

## Supplementary Information

**Additional file 1**: **Figure S1** Embryonic development stages and hatching of Necator americanus, Ancylostoma ceylanicum, and Heligmosomoides polygyrus eggs. **Figure S2** Viability of Necator americanus, Ancylostoma ceylanicum, and Heligmosomoides polygyrus eggs incubated within PBS, HBSS, and RPMI at room temperature. **Figure S3** Hatching of unembryonated and embryonated Heligmosomoides polygyrus eggs. **Figure S4** Embryonic development of freshly isolated Heligmosomoides polygyrus eggs incubated at 4 °C. *Figure S5* Hatching of Heligmosomoides polygyrus eggs at room temperature and 4 °C. **Figure S6** Observed delays in hatching of embryonated Heligmosomoides polygyrus eggs within media of increasing NaCl concentrations. **Figure S7** Viability of Heligmosomoides polygyrus eggs in media of increasing NaCl concentrations and various acidities at room temperature. **Figure S8** Abnormal appearing embryonated Heligmosomoides polygyrus eggs observed within hyperosmolar and pH 2 media. **Figure S9** Chemical structures of the evaluated anthelminthics. **Figure S10** In vitro concentration-response curve and EC50 value determination among egg-hatch assays. **Table S1** Compound concentration range for in vitro EC50 determination.

## Data Availability

All data generated or analysed during this study are included in this published article and its supplementary information files.
