# Supplementary material for: Development of a hookworm egg hatching assay to determine the ovicidal effects of anthelminthics
Source: Parasit Vectors. 2023 May 4;16:157. doi: 10.1186/s13071-023-05771-8 (PMC10161531; doi:10.1186/s13071-023-05771-8)
Supplement: Supplementary file 1 — Additional file 1: Figure S1 Embryonic development stages and hatching of Necator americanus, Ancylostoma ceylanicum, and Heligmosomoides polygyrus eggs. Figure S2 Viability of Necator americanus, Ancylostoma ceylanicum, and Heligmosomoides polygyrus eggs incubated within PBS, HBSS, and RPMI at room temperature. Figure S3 Hatching of unembryonated and embryonated Heligmosomoides polygyrus eggs. Figure S4 Embryonic development of freshly isolated Heligmosomoides polygyrus eggs incubated at 4 °C. Figure S5 Hatching of Heligmosomoides polygyrus eggs at room temperature and 4 °C. Figure S6 Observed delays in hatching of embryonated Heligmosomoides polygyrus eggs within media of increasing NaCl concentrations. Figure S7 Viability of Heligmosomoides polygyrus eggs in media of increasing NaCl concentrations and various acidities at room temperature. Figure S8 Abnormal appearing embryonated Heligmosomoides polygyrus eggs observed within hyperosmolar and pH 2 media. Figure S9 Chemical structures of the evaluated anthelminthics. Figure S10 In vitro concentration-response curve and EC50 value determination among egg-hatch assays. Table S1 Compound concentration range for in vitro EC50 determination. [file 13071_2023_5771_MOESM1_ESM.docx]

**Development of a hookworm egg hatching assay for determination of ovicidal effects of anthelminthics**

**Supplemental Material – Additional File 1**

*Erin Easland^1,2 ‡^, Stefan Biendl^1,2 ‡^ and Jennifer Keiser^1,2^**

^‡^E. Easland, S. Biendl have contributed equally (joint first authorship).

*Corresponding author. E-mail: jennifer.keiser@swisstph.ch

^1^E. Easland, S. Biendl, Prof. J. Keiser

Swiss Tropical and Public Health Institute

Department of Medical Parasitology and Infection Biology

Kreuzstrasse 2, CH-4123 Allschwil, Switzerland

^2^E. Easland, S. Biendl, Prof. J. Keiser

University of Basel

CH-4003, Basel, Switzerland

Erin Easland: https://orcid.org/0000-0002-9594-2863

Stefan Biendl: https://orcid.org/0000-0002-7859-5601

Jennifer Keiser: http://orcid.org/0000-0003-0290-3521

**Keywords**

anthelminthics, drug discovery, hookworm, egg hatching, *Heligmosomoides polygyrus*, *Necator americanus*, *Ancylostoma duodenale*


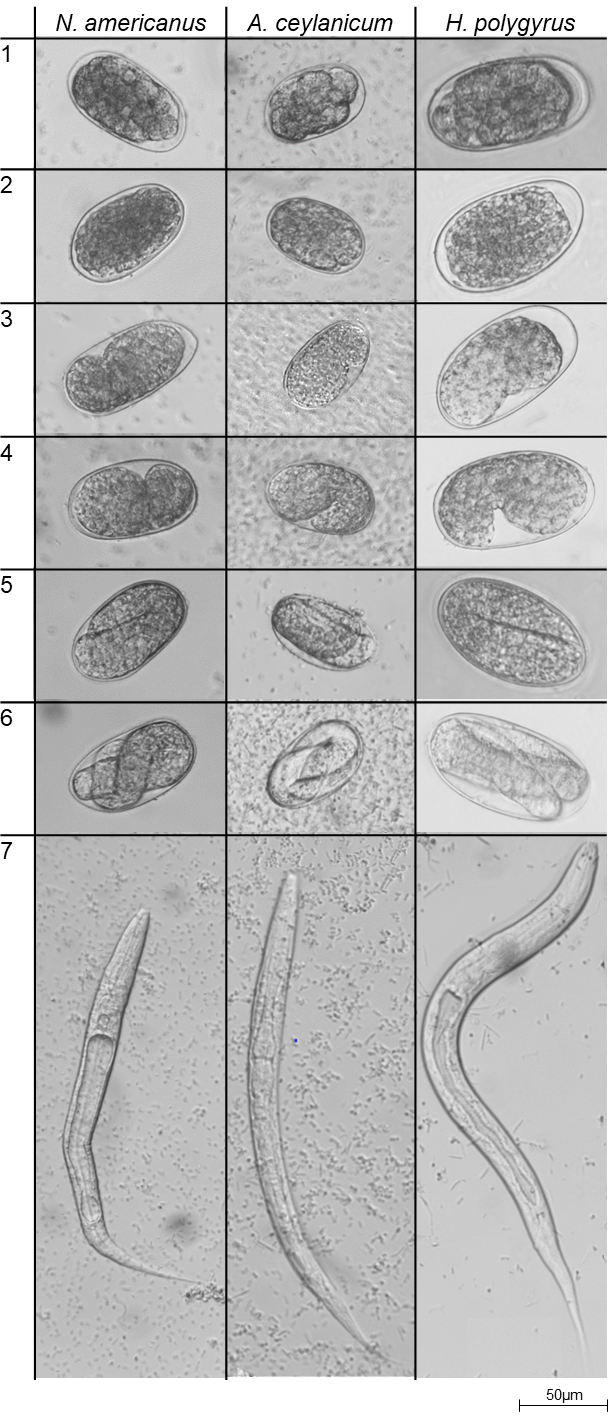


**Figure S1**: See next page for caption.

**Figure S1**: Embryonic development stages and hatching of *N. americanus*, *A. ceylanicum*, and *H. polygyrus* eggs. Initially, a mass of blastomeres forms in eggs (1) that further mature to a smoother, more homogenous form (2). Earliest stages of embryonation are displayed by contraction and slight indentation of the oval shaped mass of blastomeres (3). Over time the indentation becomes deeper (4), leading to a form with a single fold with resemblance of a cylindrical larva (5). The embryo continues to thin and elongate into the larval form within the eggshell (6). After complete embryonation the larva is clearly present within the eggshell (7). Photographs of hookworm eggs and larvae were captured under an inverted transmitted-light microscope at 32x magnification.

**
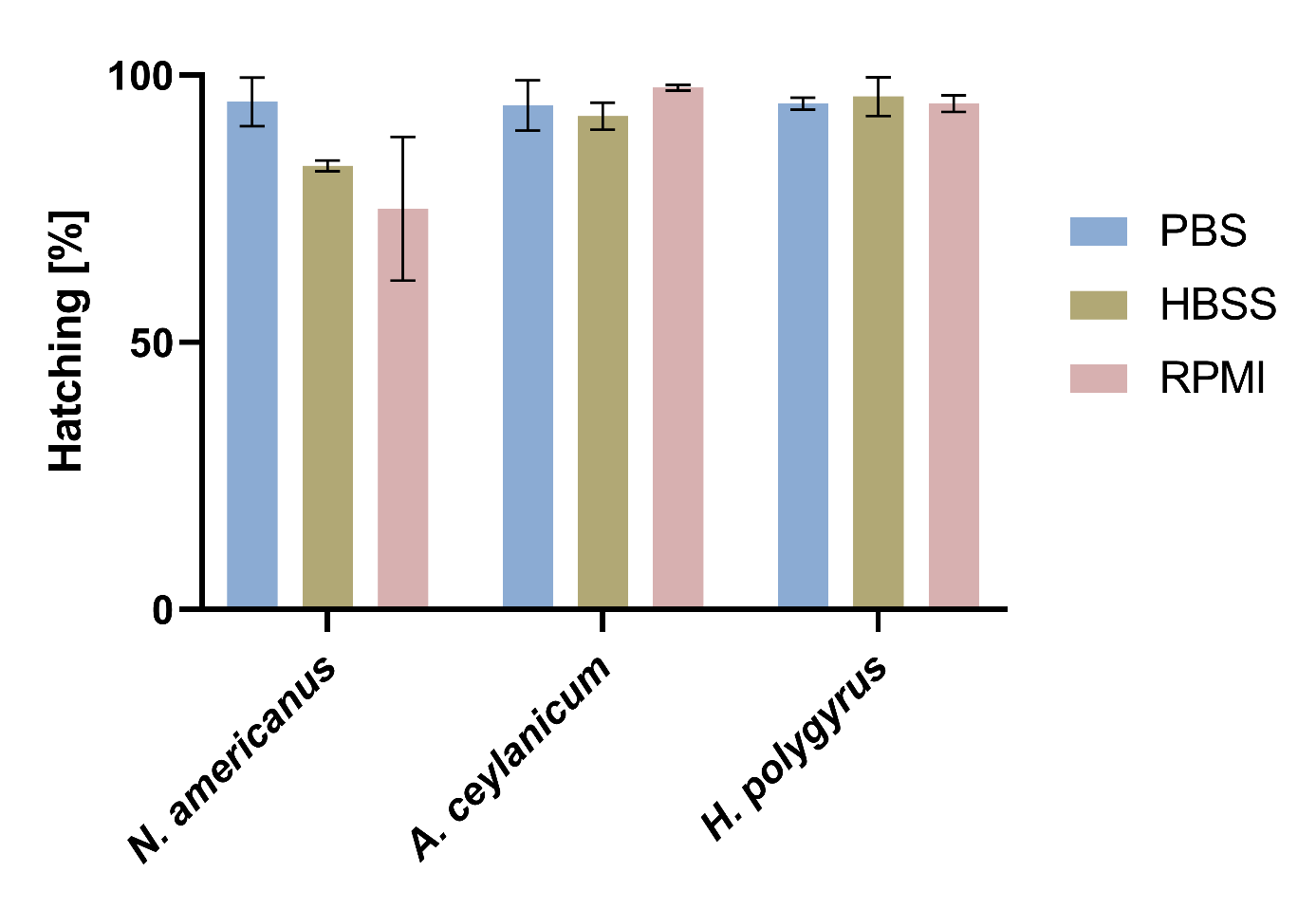
**

**Figure S2:** Viability of *N. americanus*, *A. ceylanicum*, and *H. polygyrus* eggs incubated within PBS, HBSS, and RPMI at room temperature. The columns display the mean percentage of eggs hatched after 34 hours in each media with error bars representing the standard deviation of the mean value.

**
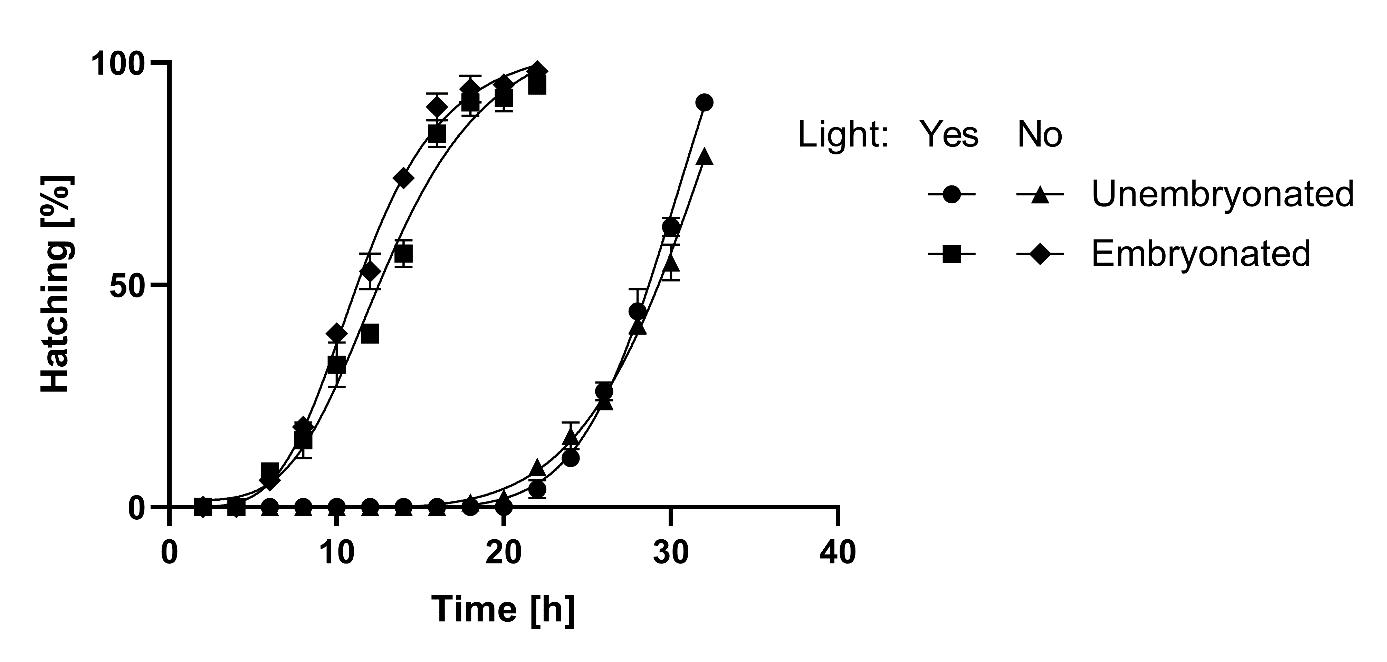
**

**Figure S3**: Hatching of unembryonated and embryonated *H. polygyrus* eggs.

The mean percent of hatched eggs was determined every 2 hours over 32 hours at room temperature with light exposure or protected from light. Error bars represent standard deviation of mean values.

**
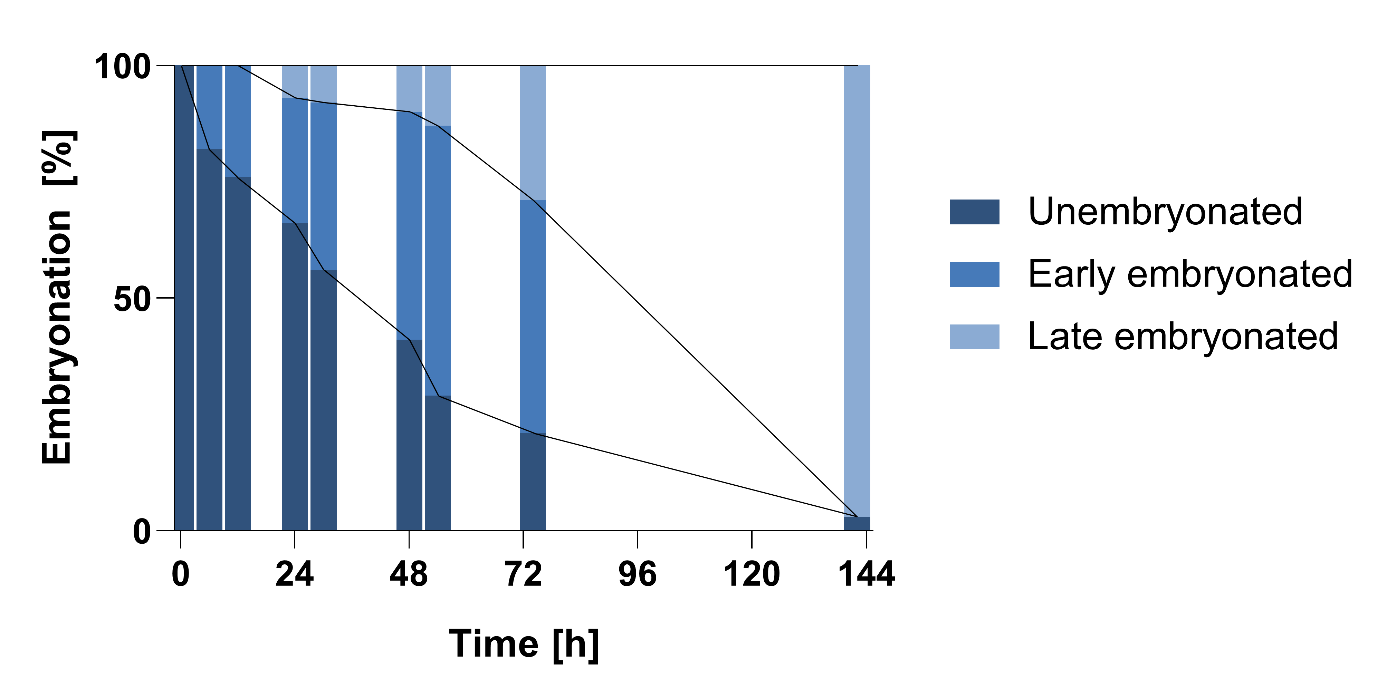
**

**Figure S4:** Embryonic development of freshly isolated *H. polygyrus* eggs incubated at 4°C. The graph displays the mean percentage of eggs showing no embryonation, early embryonation, and late embryonation over time.

**
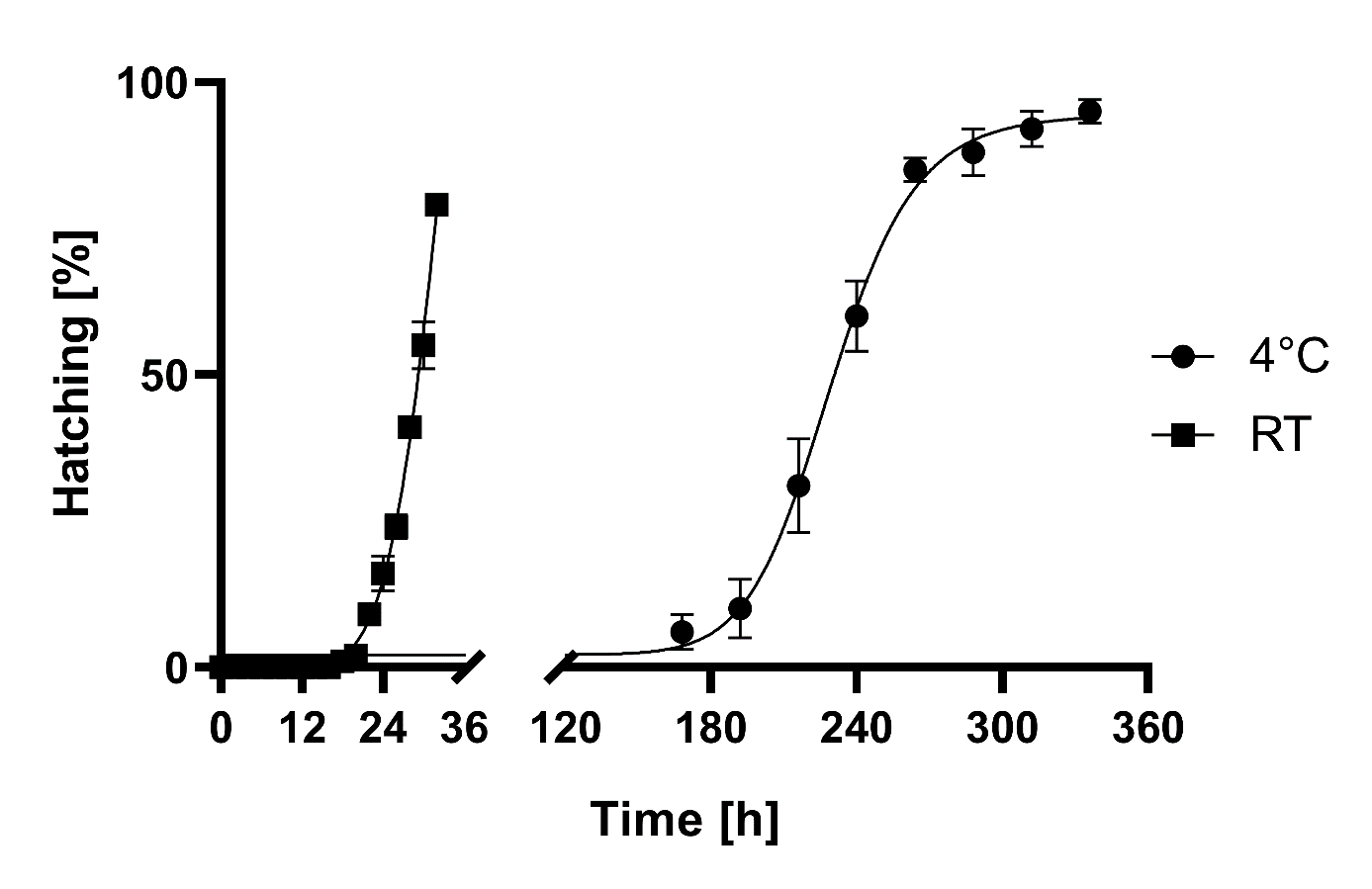
**

**Figure S5:** Hatching of *H. polygyrus* eggs at room temperature and 4°C. The graph displays the mean percentage of eggs hatched over 32 hours at room temperature and 336 hours at 4°C with error bars representing the standard deviation of mean values.

**
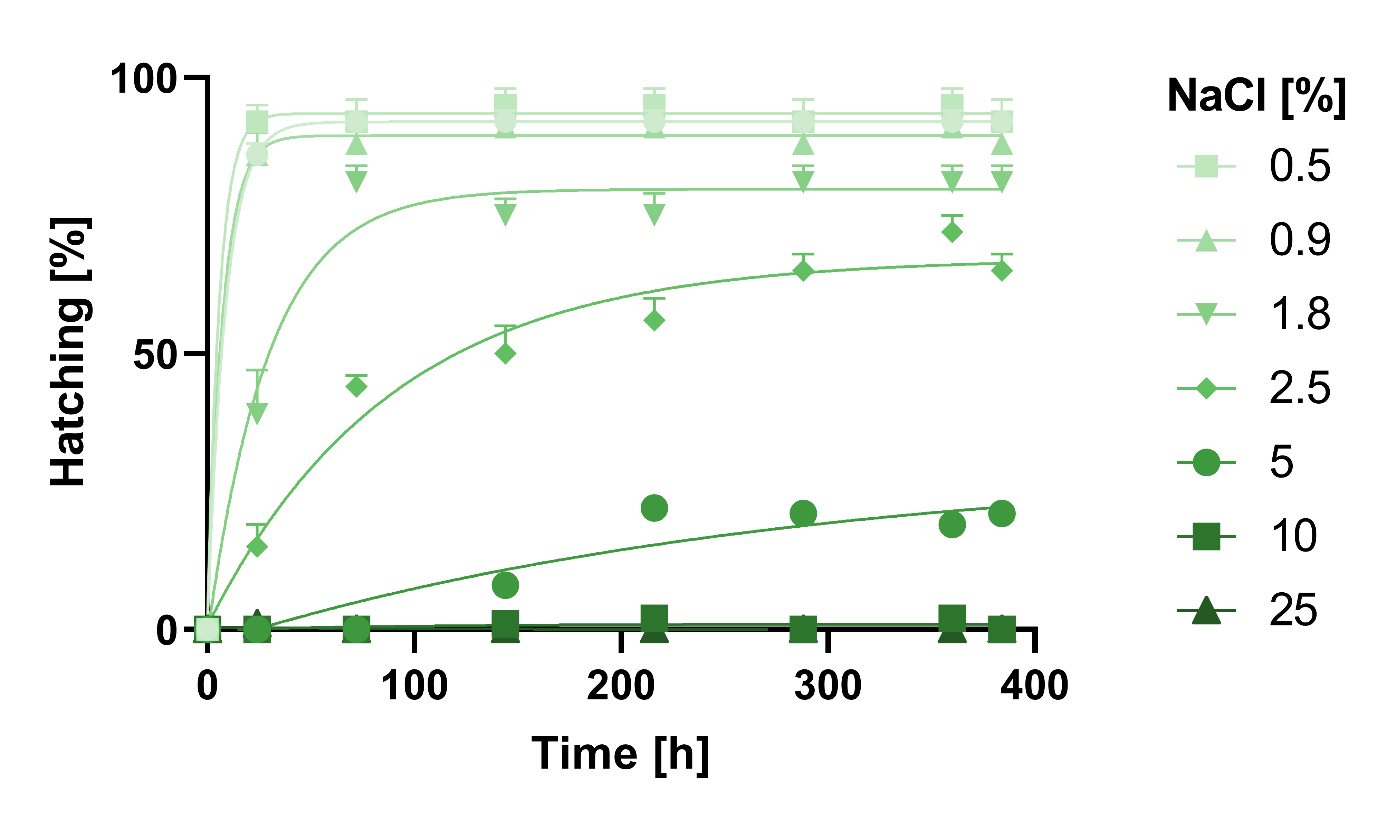
**

**Figure S6:** Observed delays in hatching of embryonated *H. polygyrus* eggs within media of increasing NaCl concentrations. The graph depicts the mean percent of hatched eggs within each media incubated at room temperature over 360 hours. Error bars represent standard deviation of mean values.


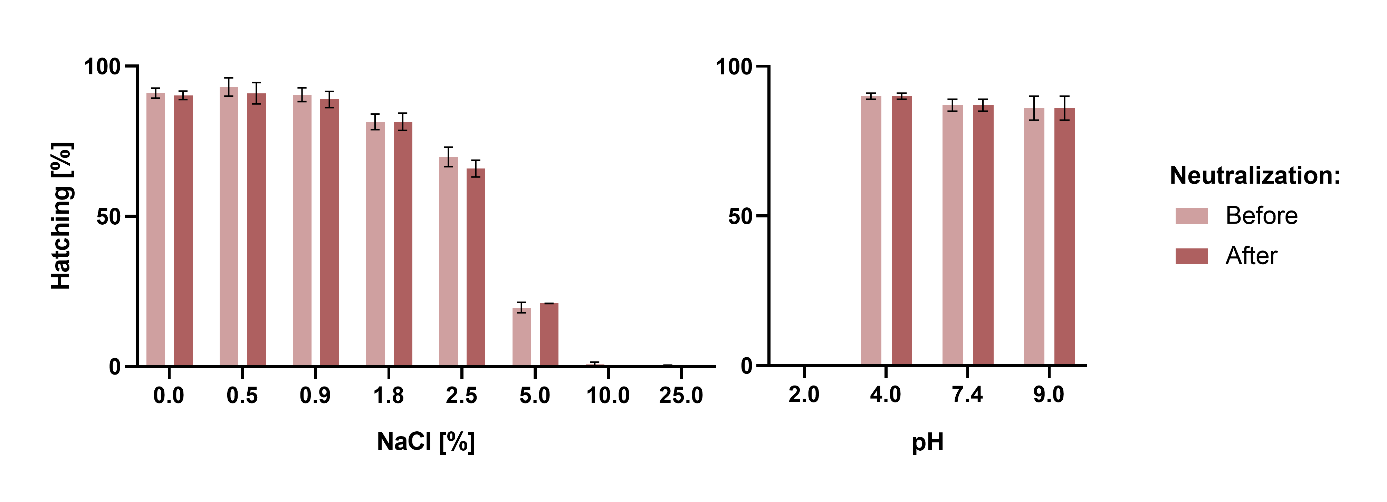


**Figure S7:** Viability of *H. polygyrus* eggs in media of increasing NaCl concentrations and various acidities at room temperature. The columns depict the mean percent of hatched eggs before (360 h) and after (384 h) neutralization of media to 0% NaCl or pH 7.4 respectively with error bars representing standard deviation of mean values.

**Figure S8:** Abnormal appearing embryonated *H. polygyrus* eggs observed within hyperosmolar (left) and pH 2 (right) media. Eggs were photographed under an inverted transmitted-light microscope with 10x magnification after 24 hours of incubation.


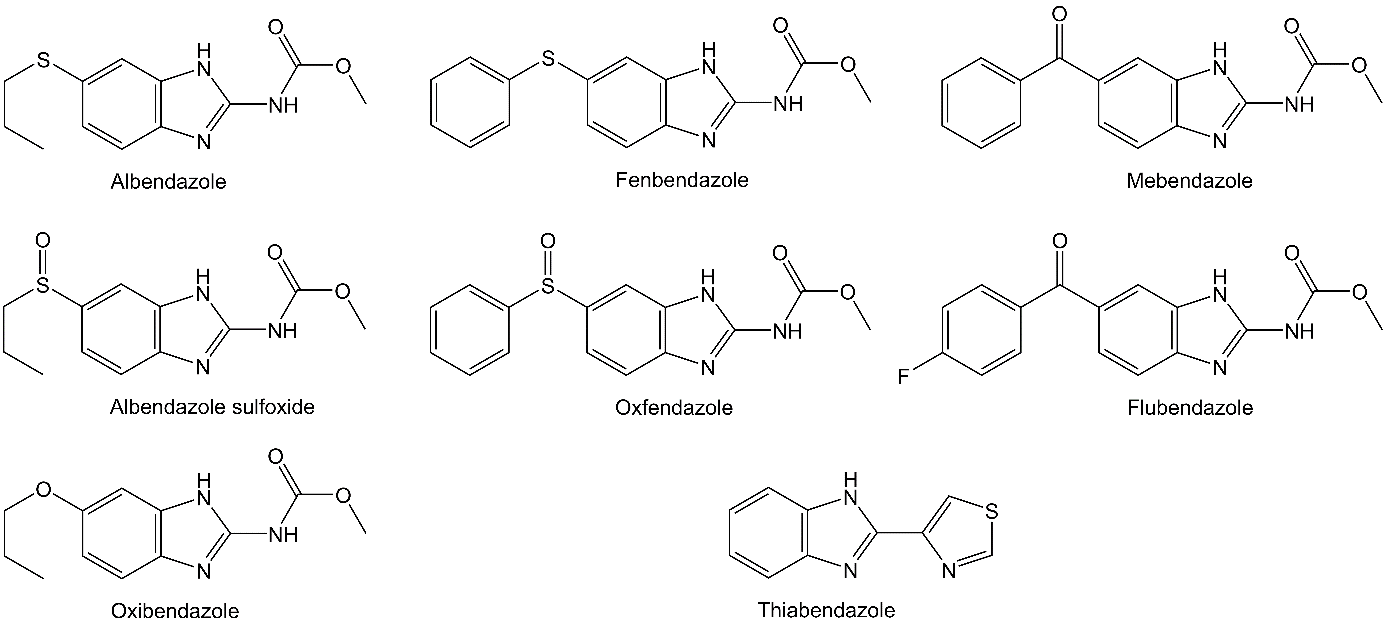


**Figure S9 A:** Chemical structures of the evaluated benzimidazole anthelminthics.


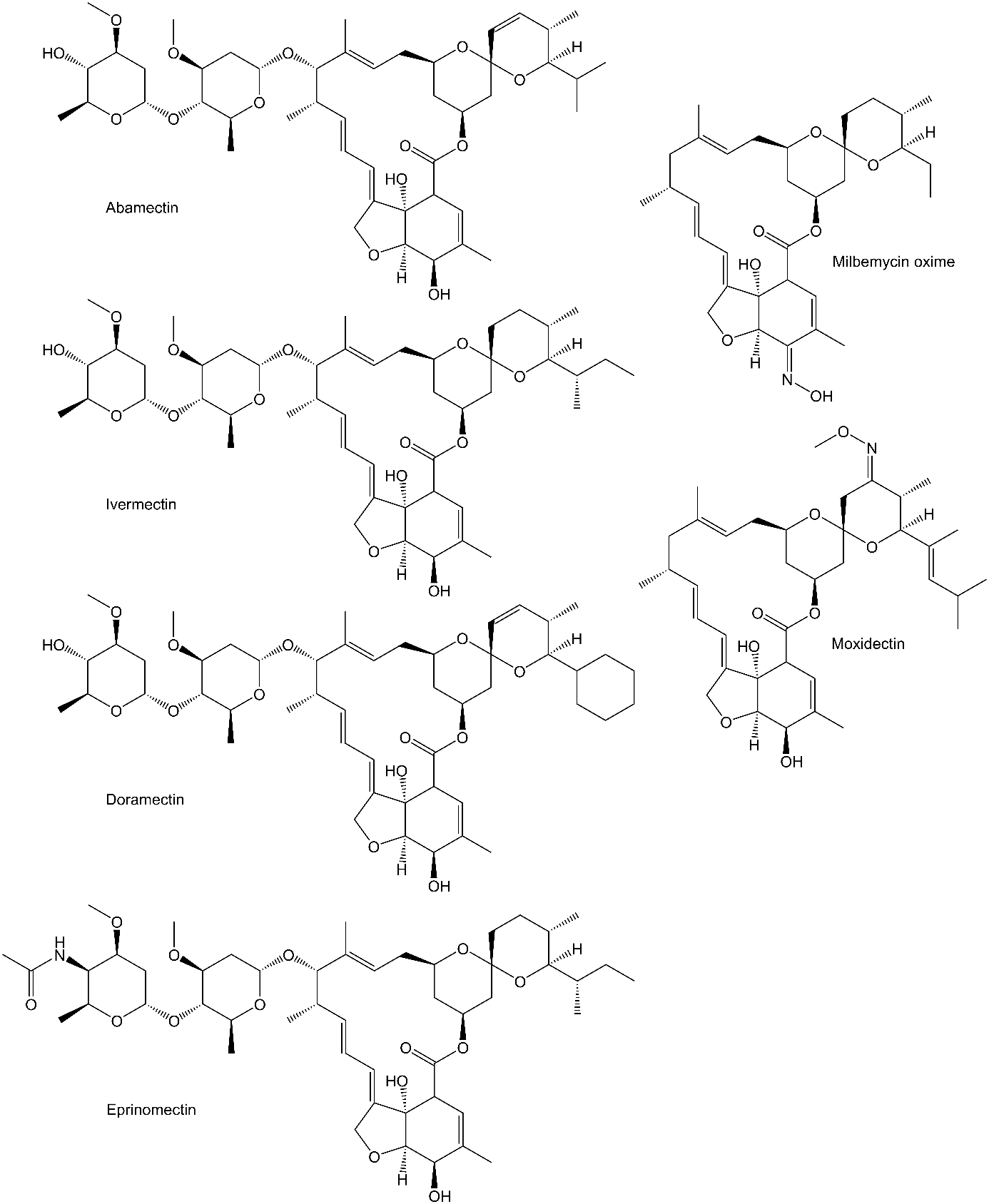


**Figure S9 B:** Chemical structures of the evaluated macrolide anthelminthics.


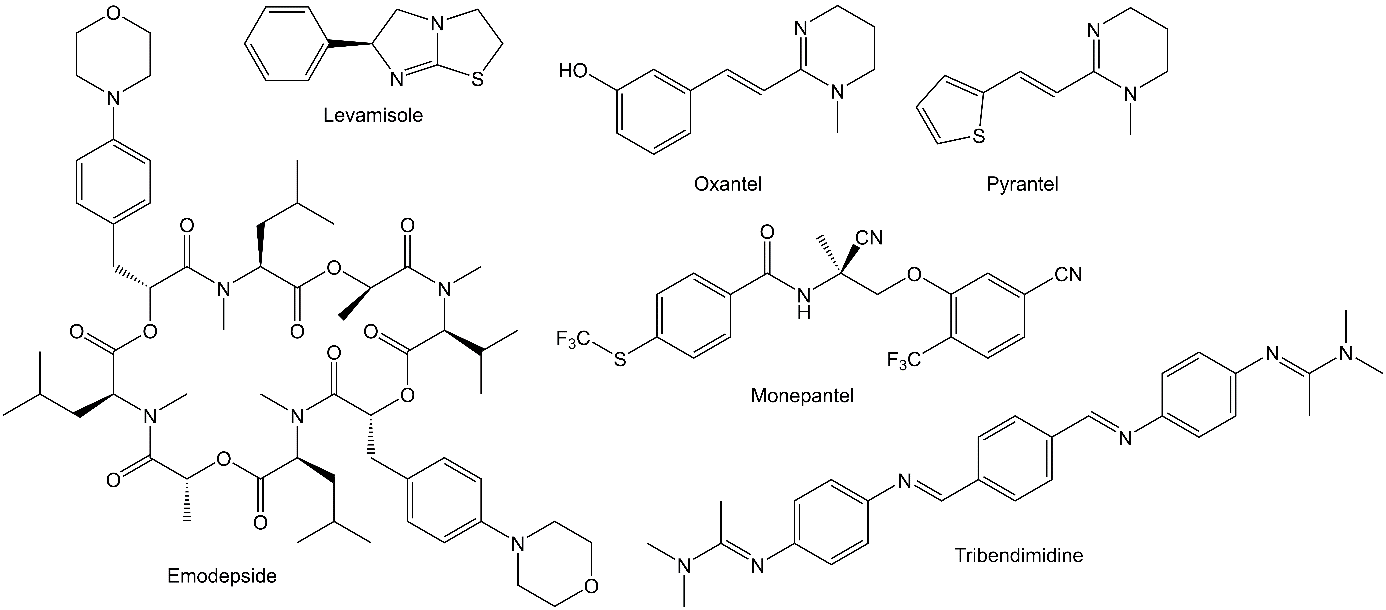


**Figure S9 C:** Chemical structures of the evaluated miscellaneous anthelminthics.

**Figure S10**: *In vitro* concentration–response curve and EC_50_ value determination among egg-hatch assays conducted with A) thiabendazole, B) albendazole, C) Oxibendazole, D) mebendazole, E) flubendazole, F) monepantel, G) levamisole, H) tribendimidine against unembryonated *H. polygyrus* (top left), embryonated *H. polygyrus* (top right), *N. americanus* (bottom left), and *A. ceylanicum* (bottom right) after 72 h of drug exposure. Each point represents the mean value of the activity and error bars represent the standard error (s.e.) of the mean. The line represents the four-parameter sigmoid function used to determine the EC_50_ value and the dashed lines show the 95% confidence interval of the fit.

**
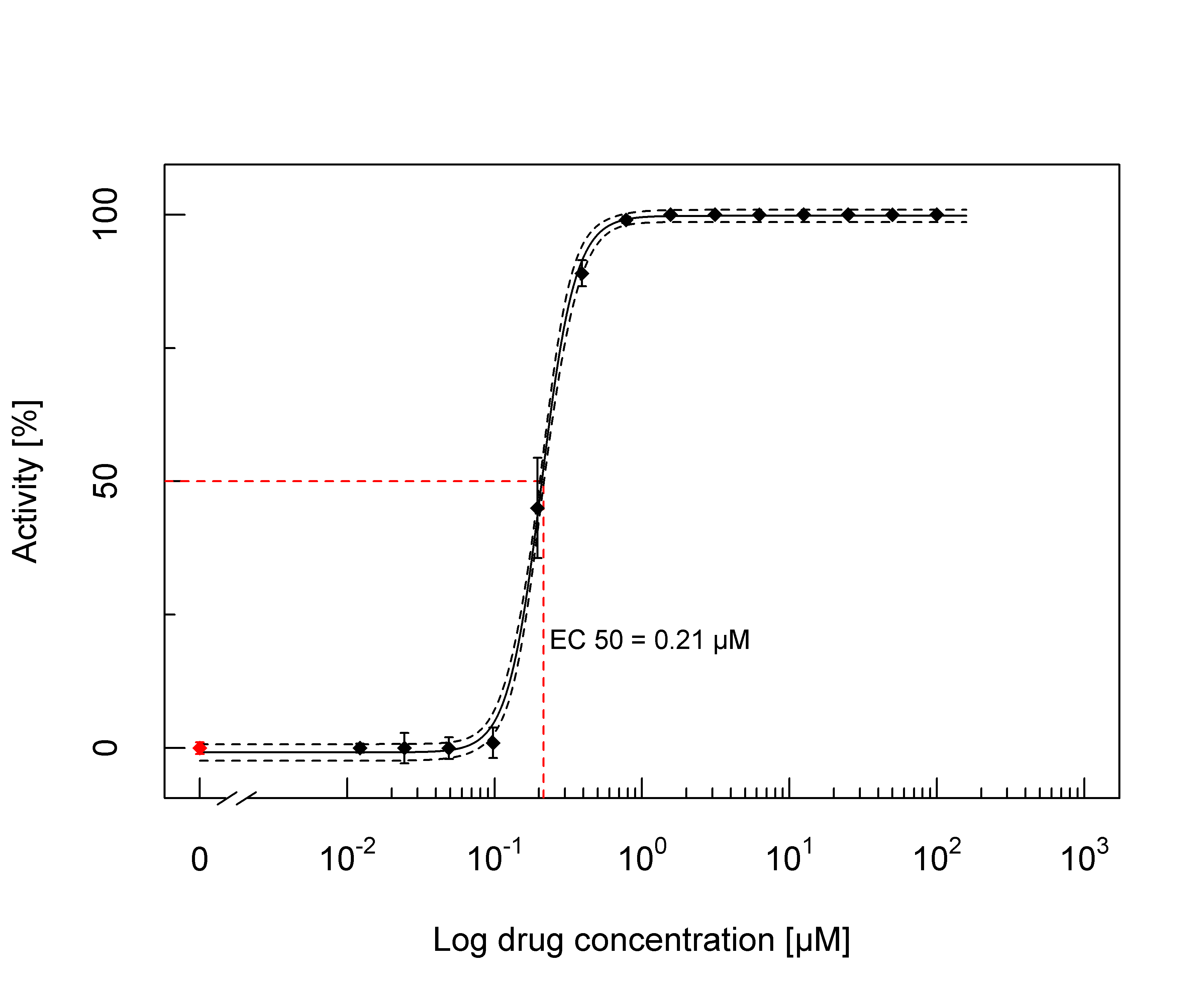

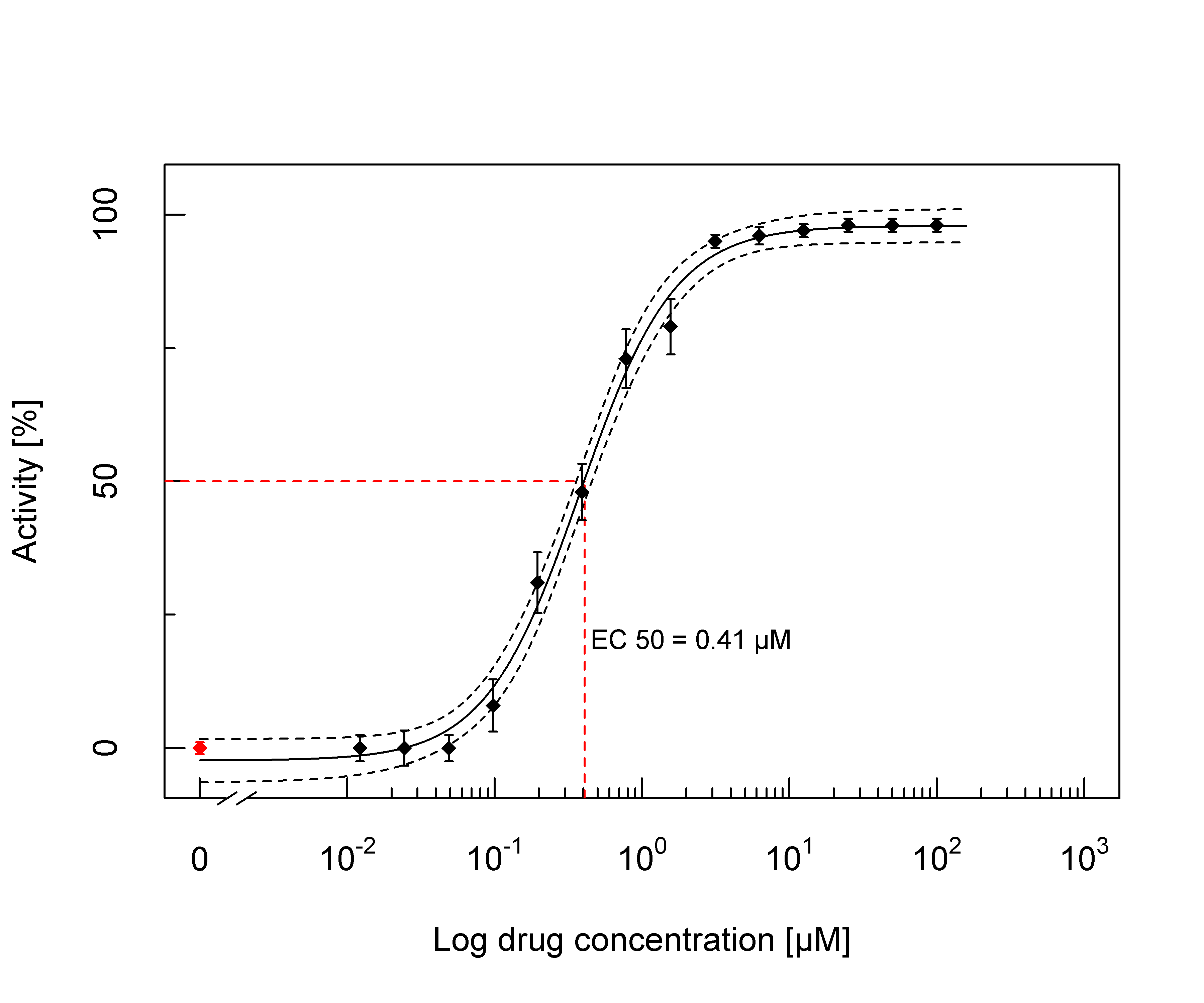

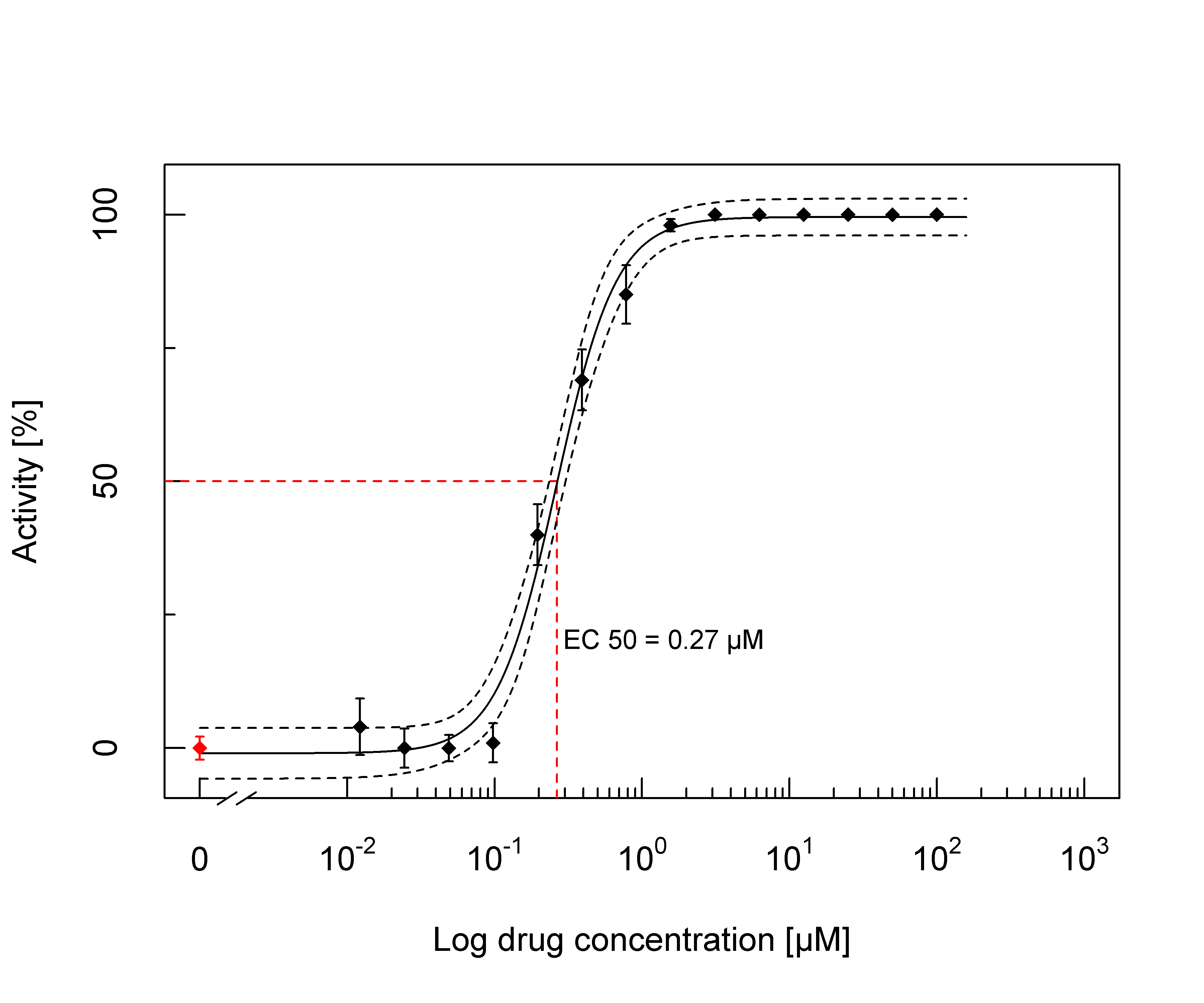

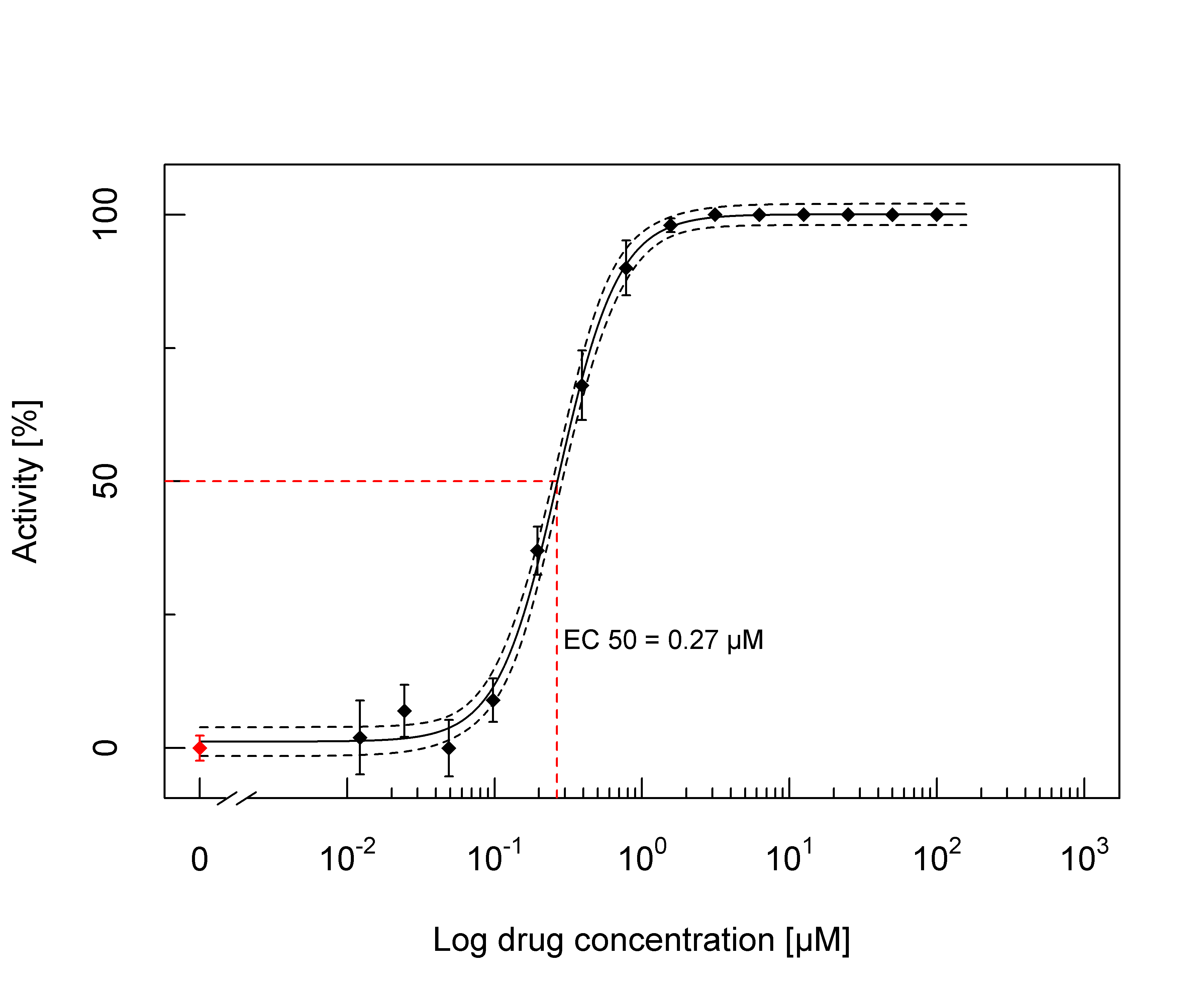
**

**Figure S10A**: Thiabendazole


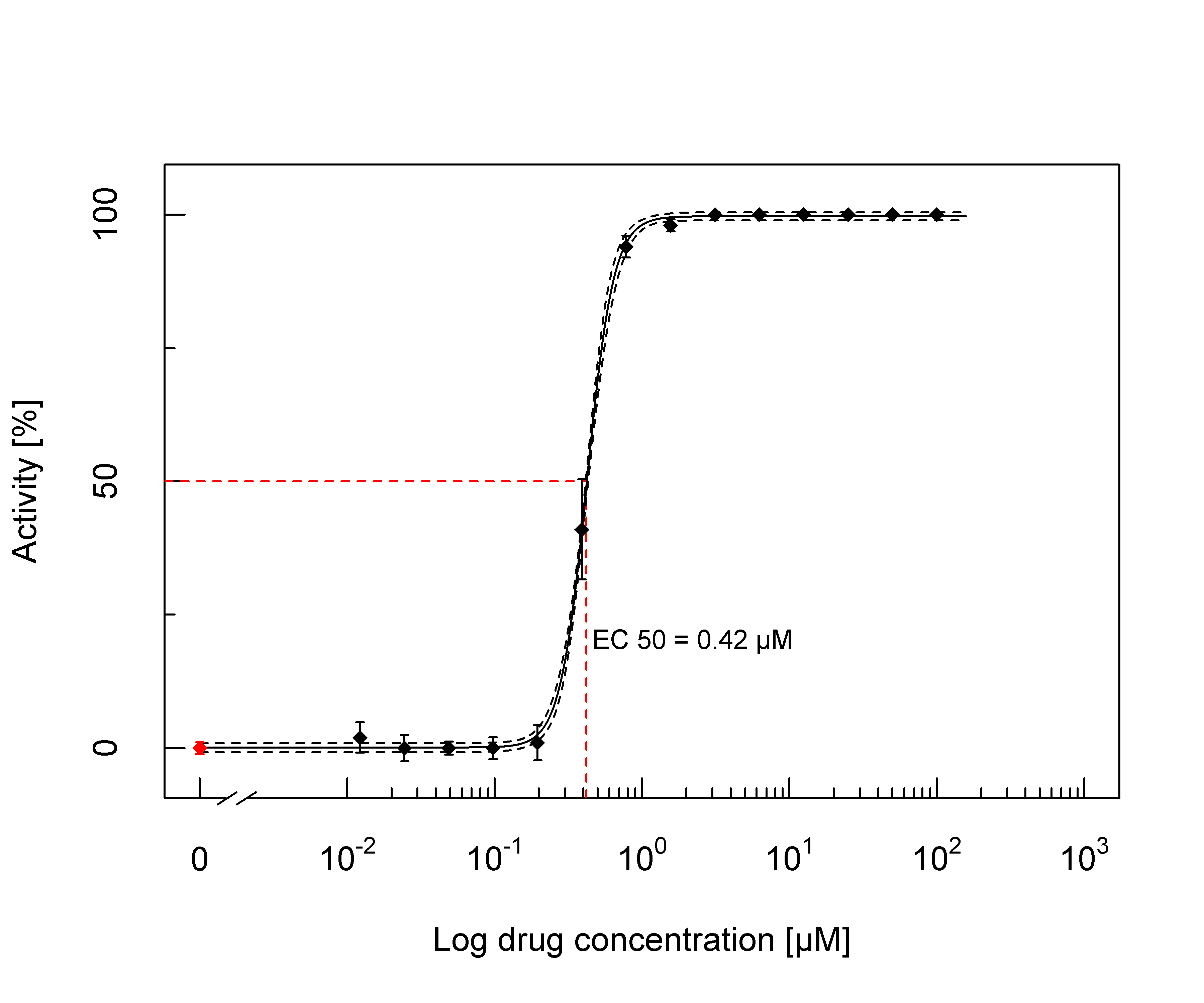

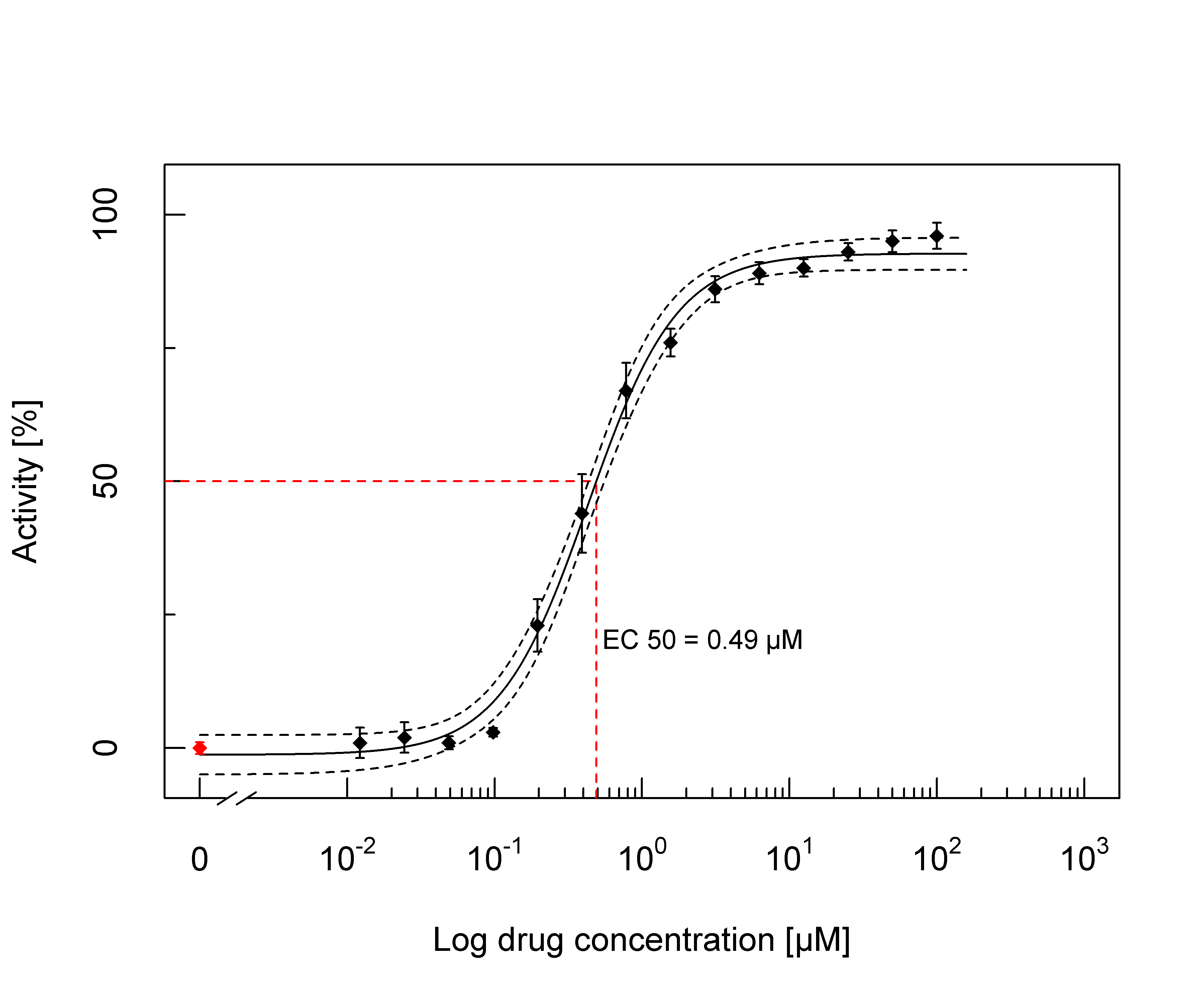

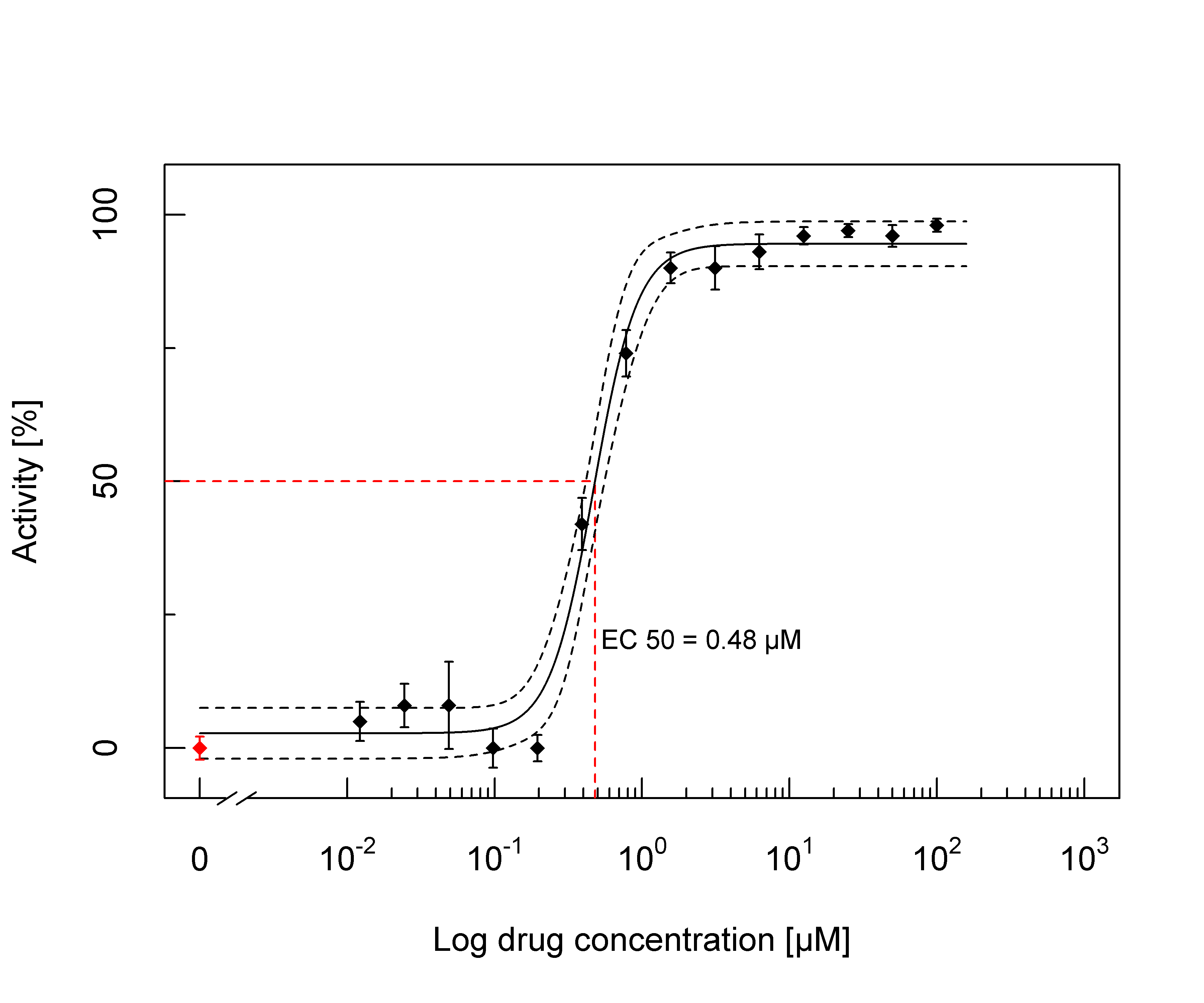

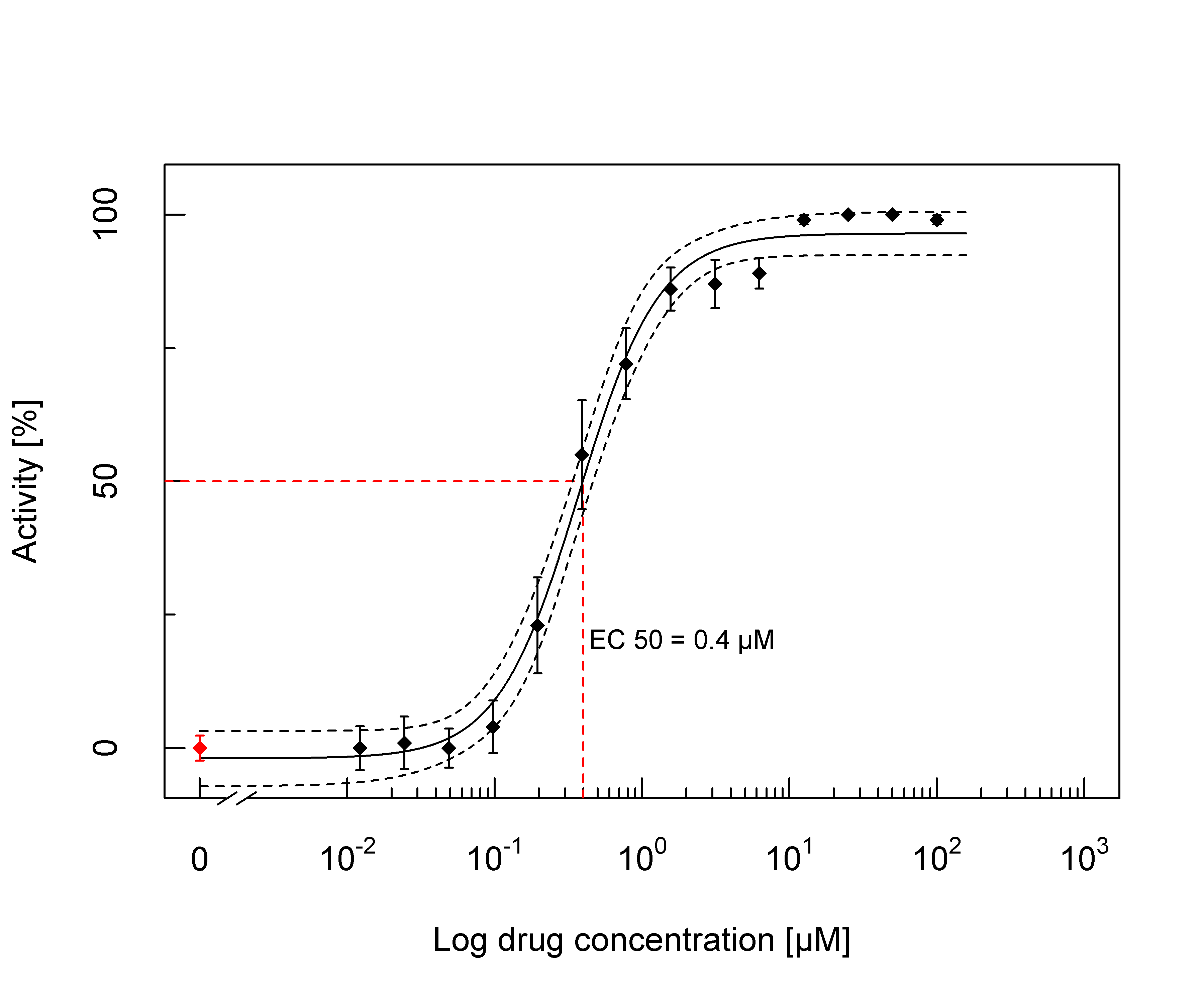


**Figure S10B**: Albendazole.


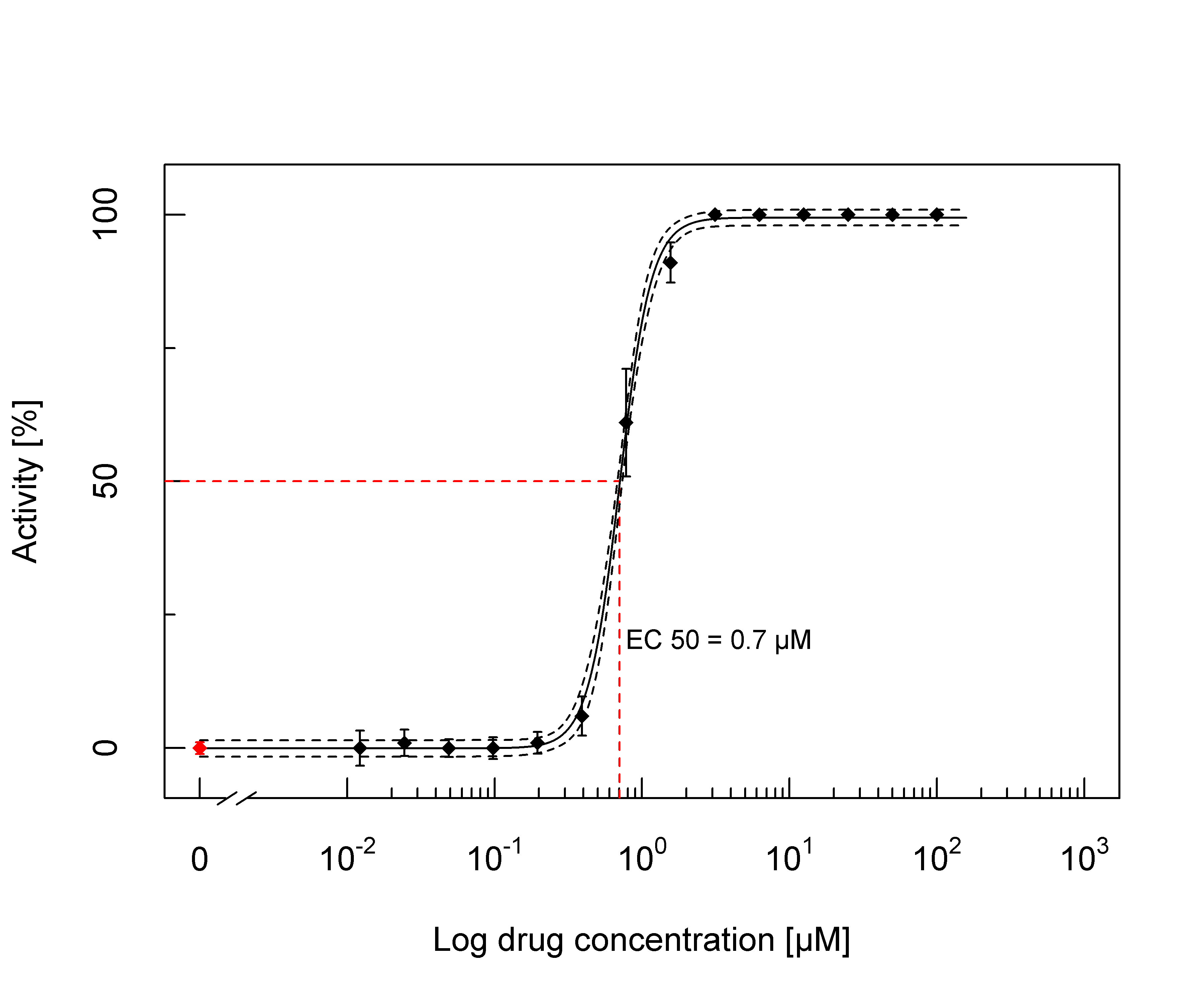

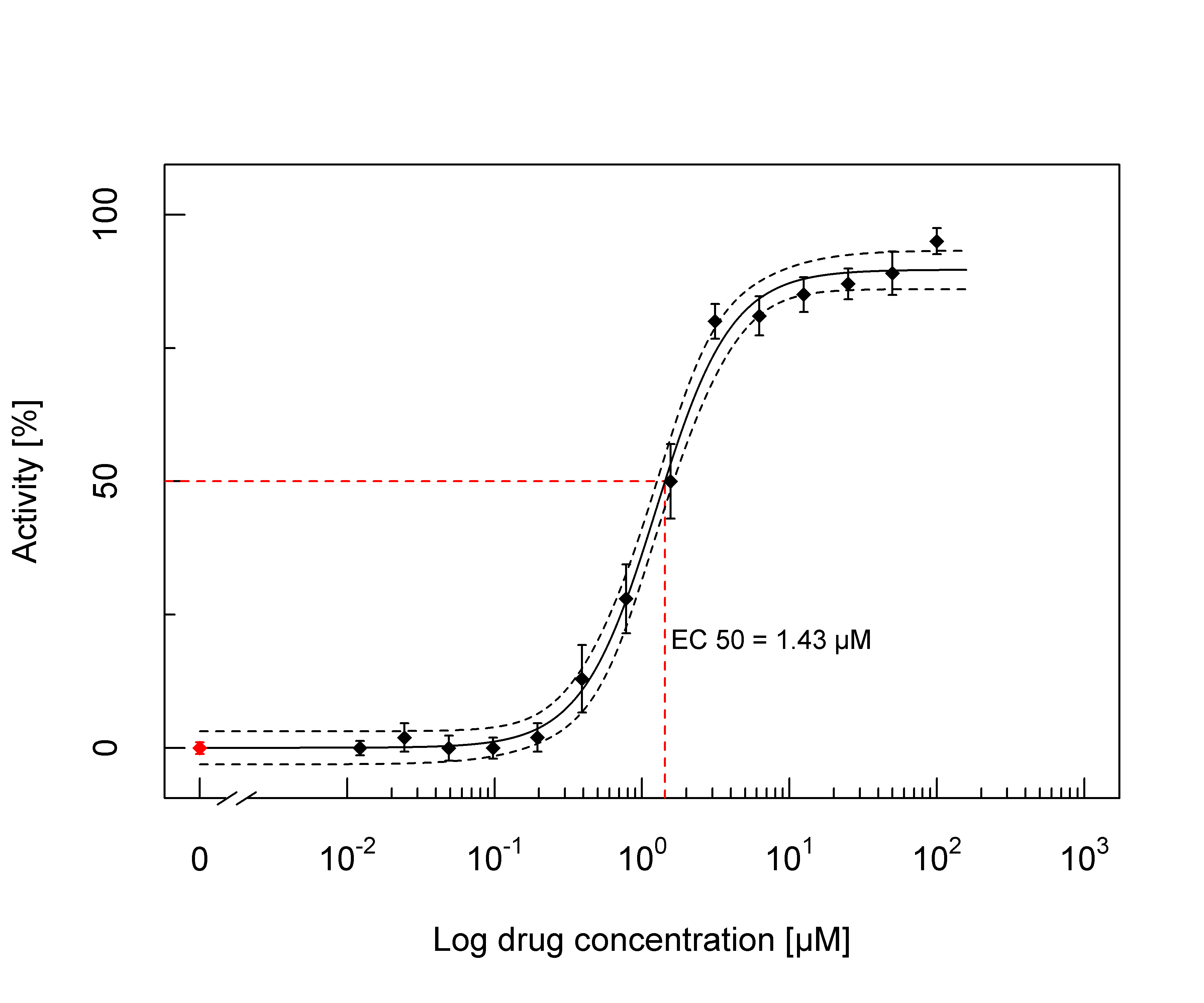

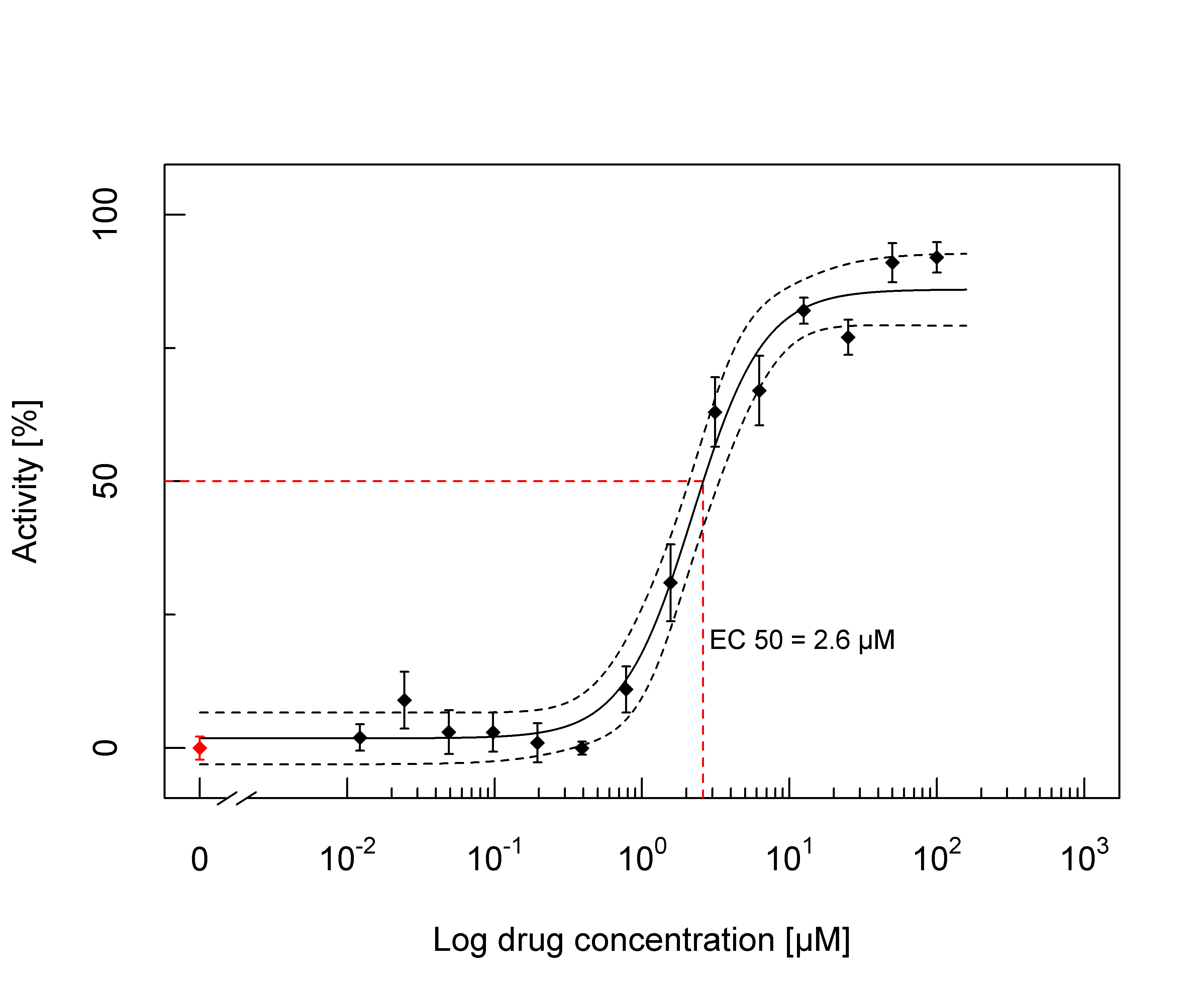

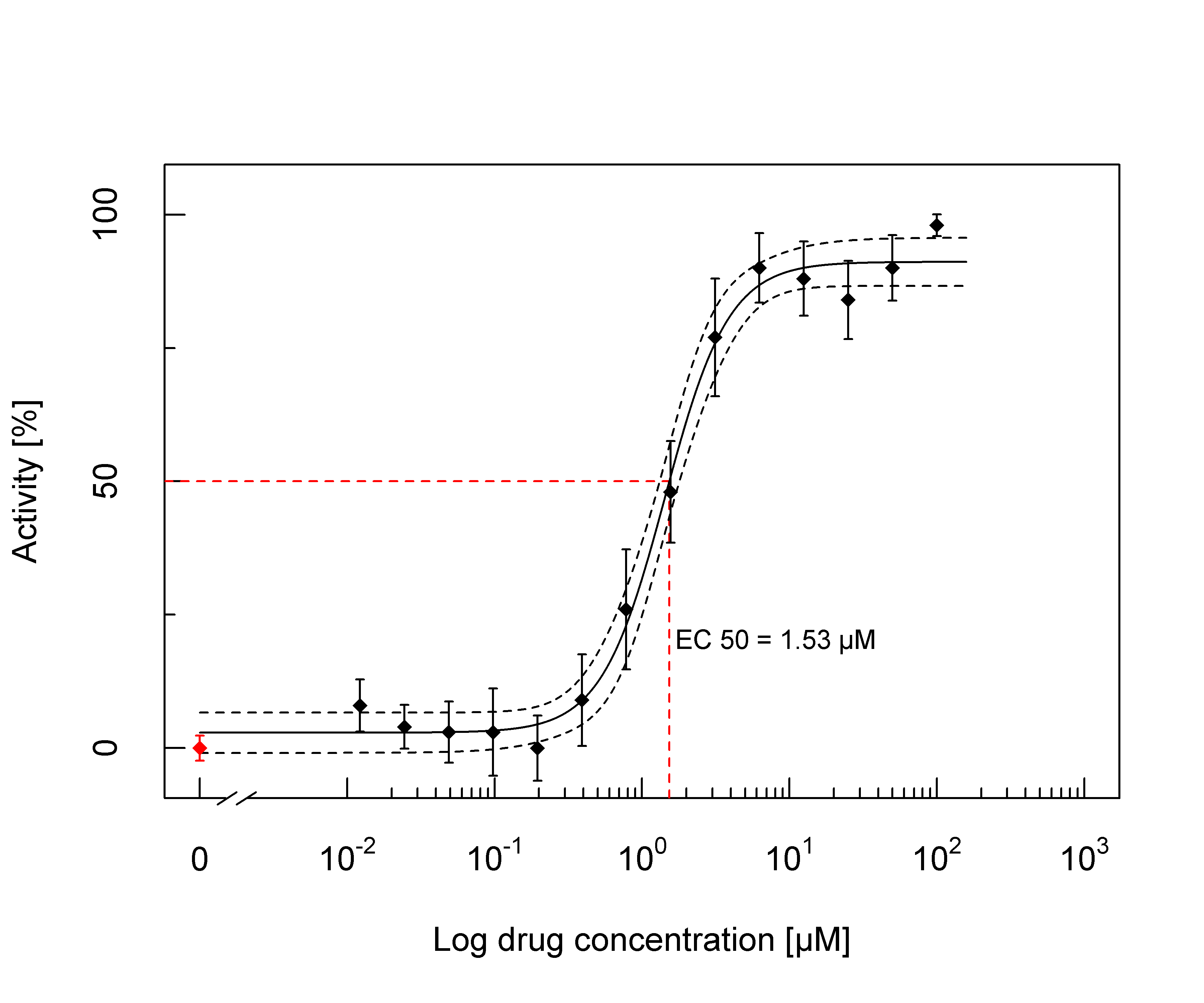


**Figure S10C:** Oxibendazole.


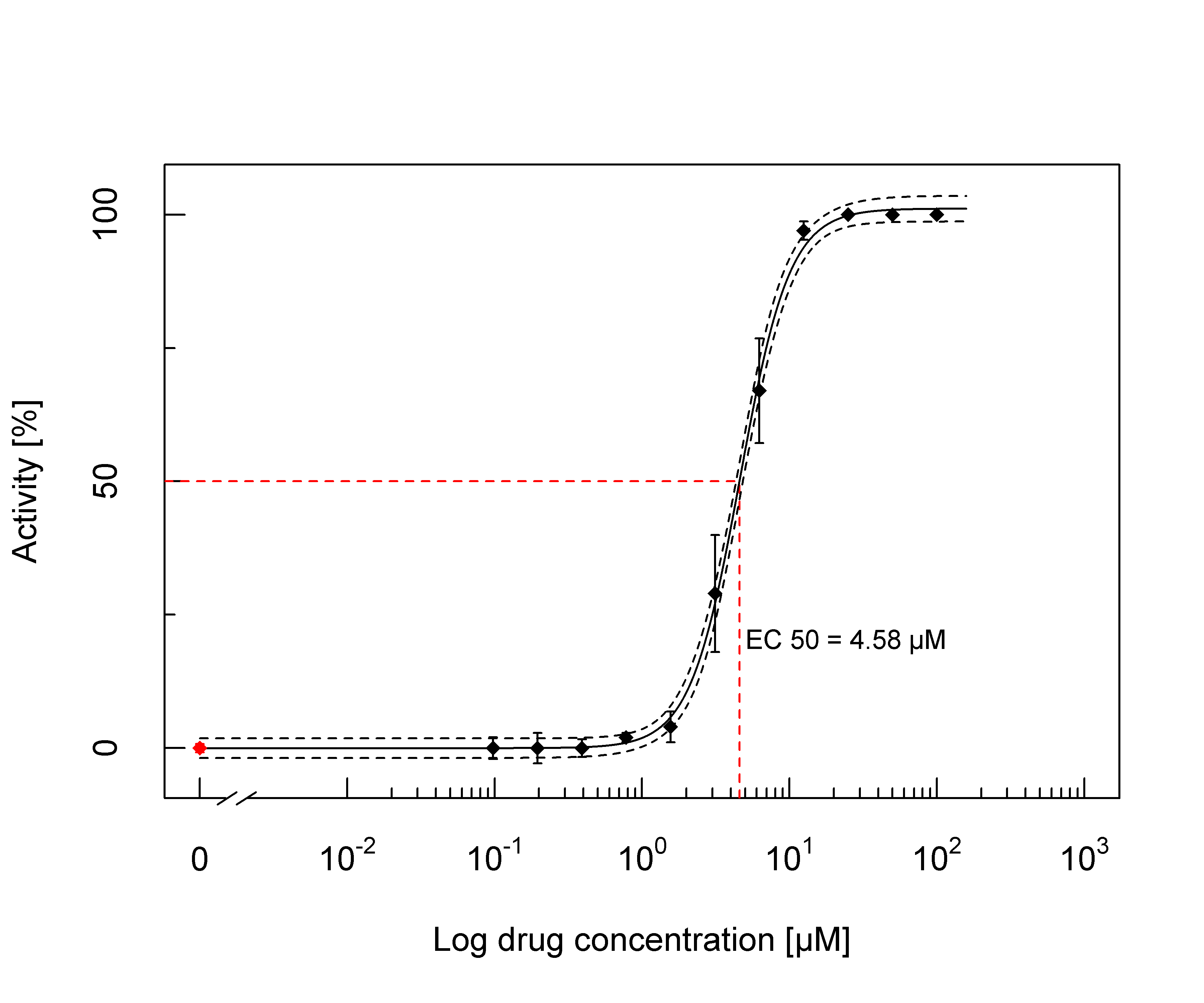

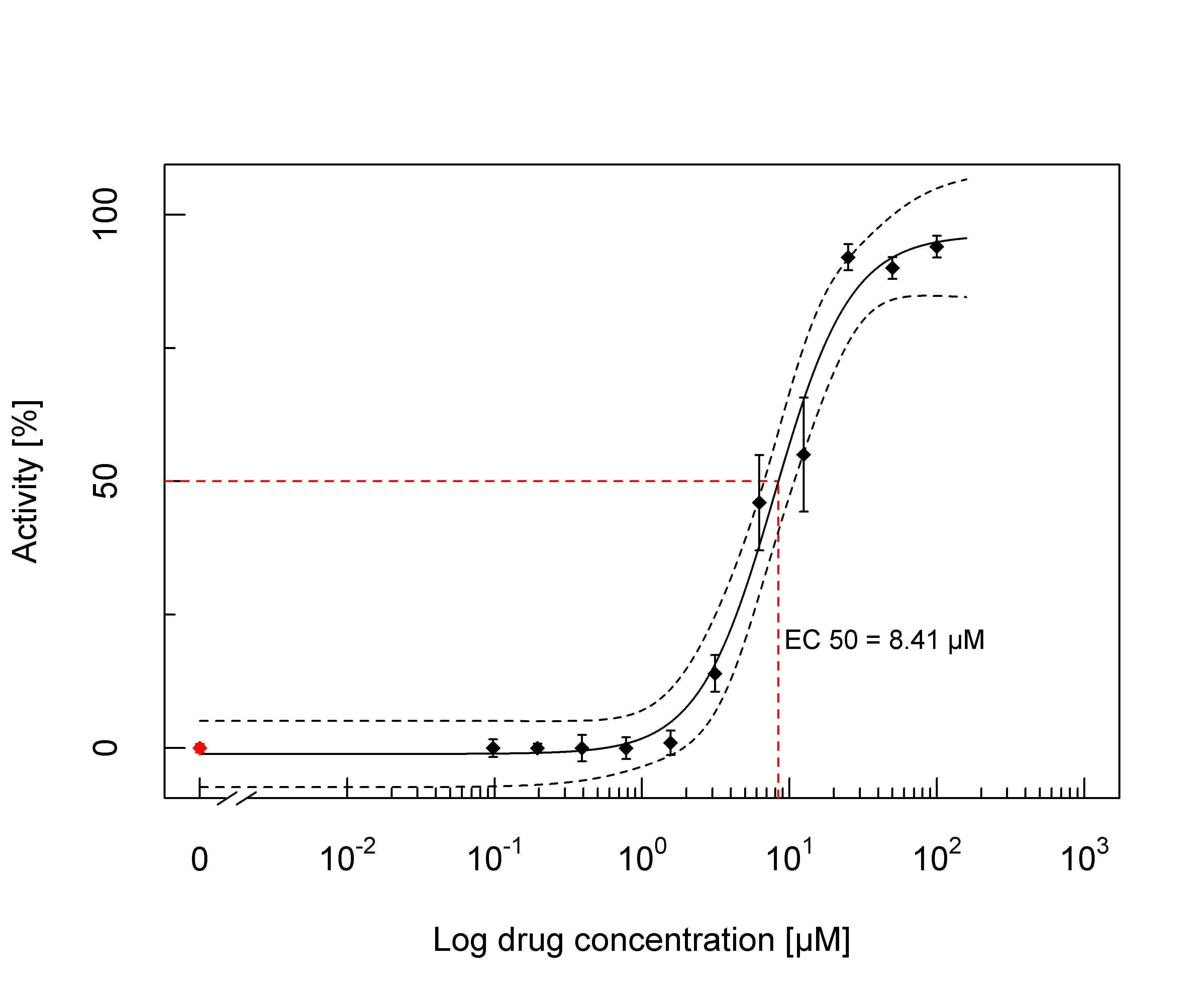


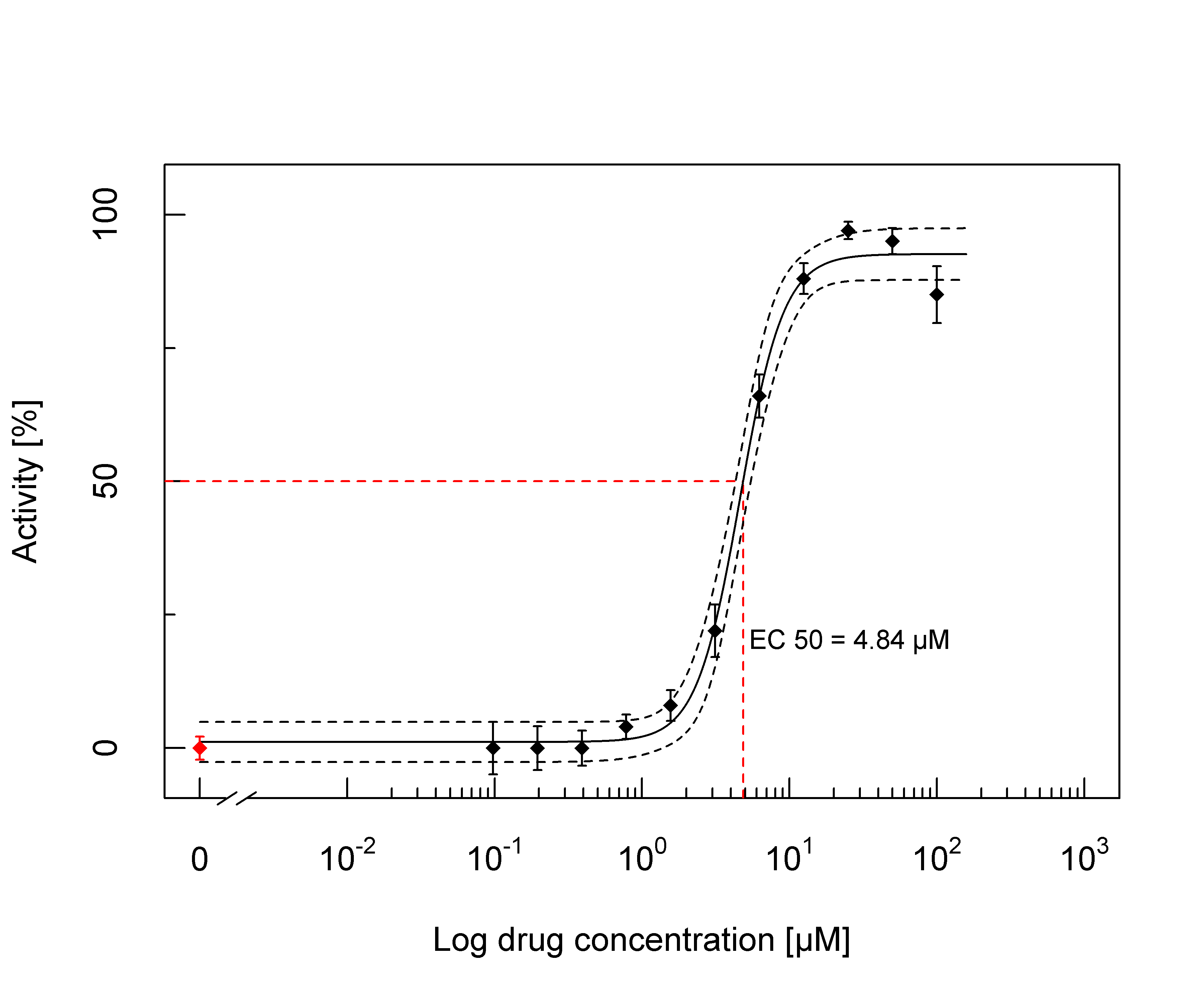

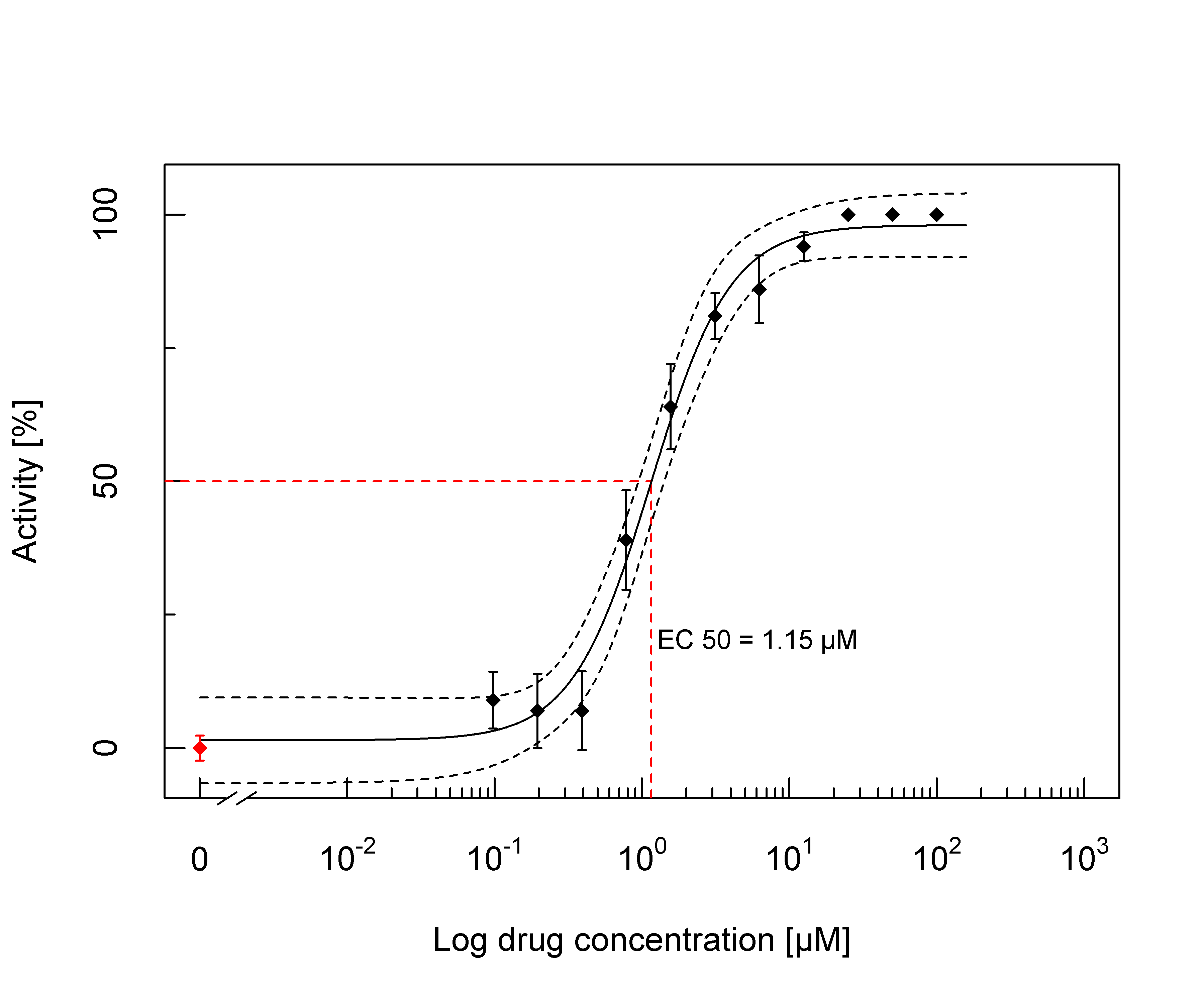


**Figure S10D:** Mebendazole.


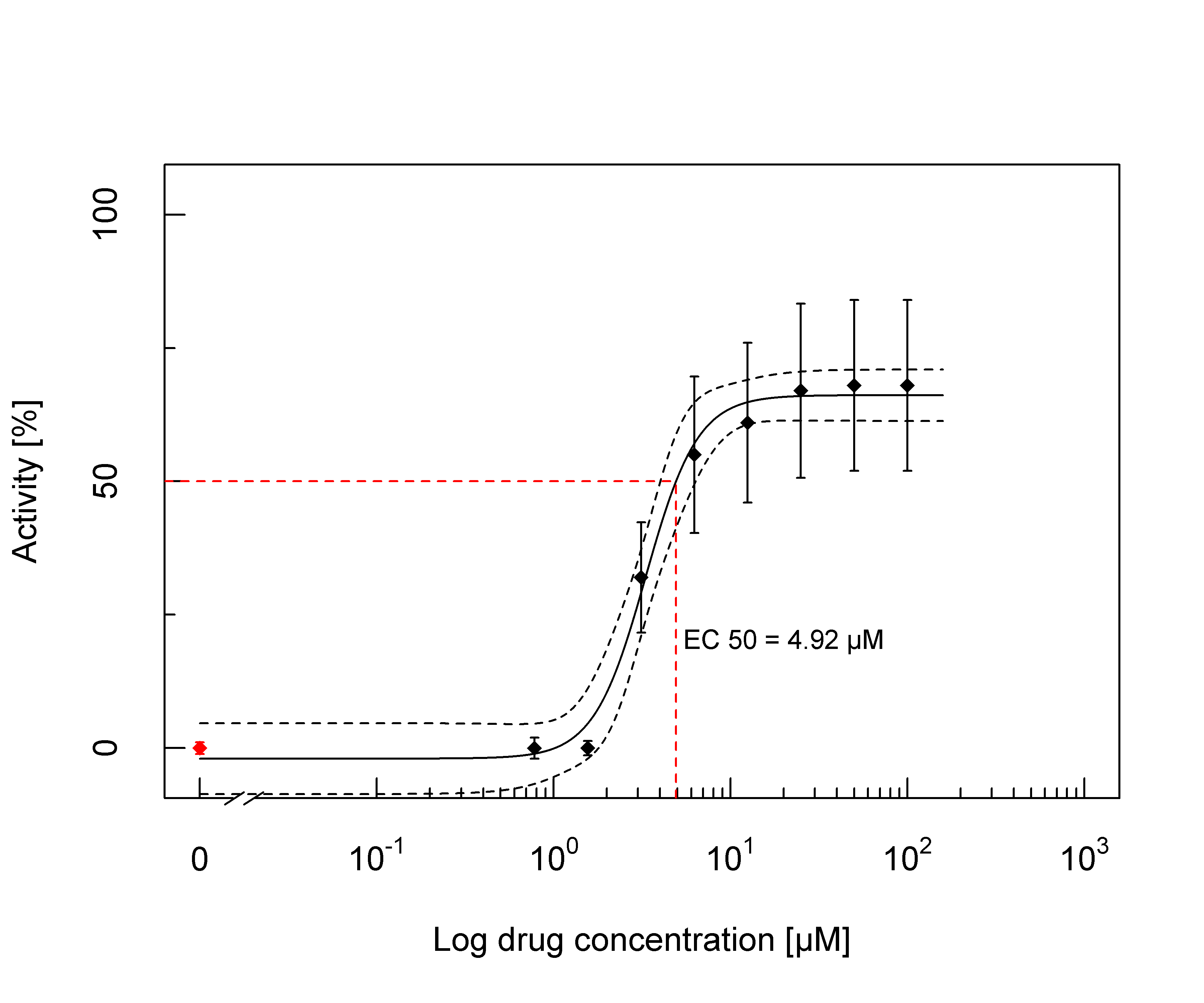

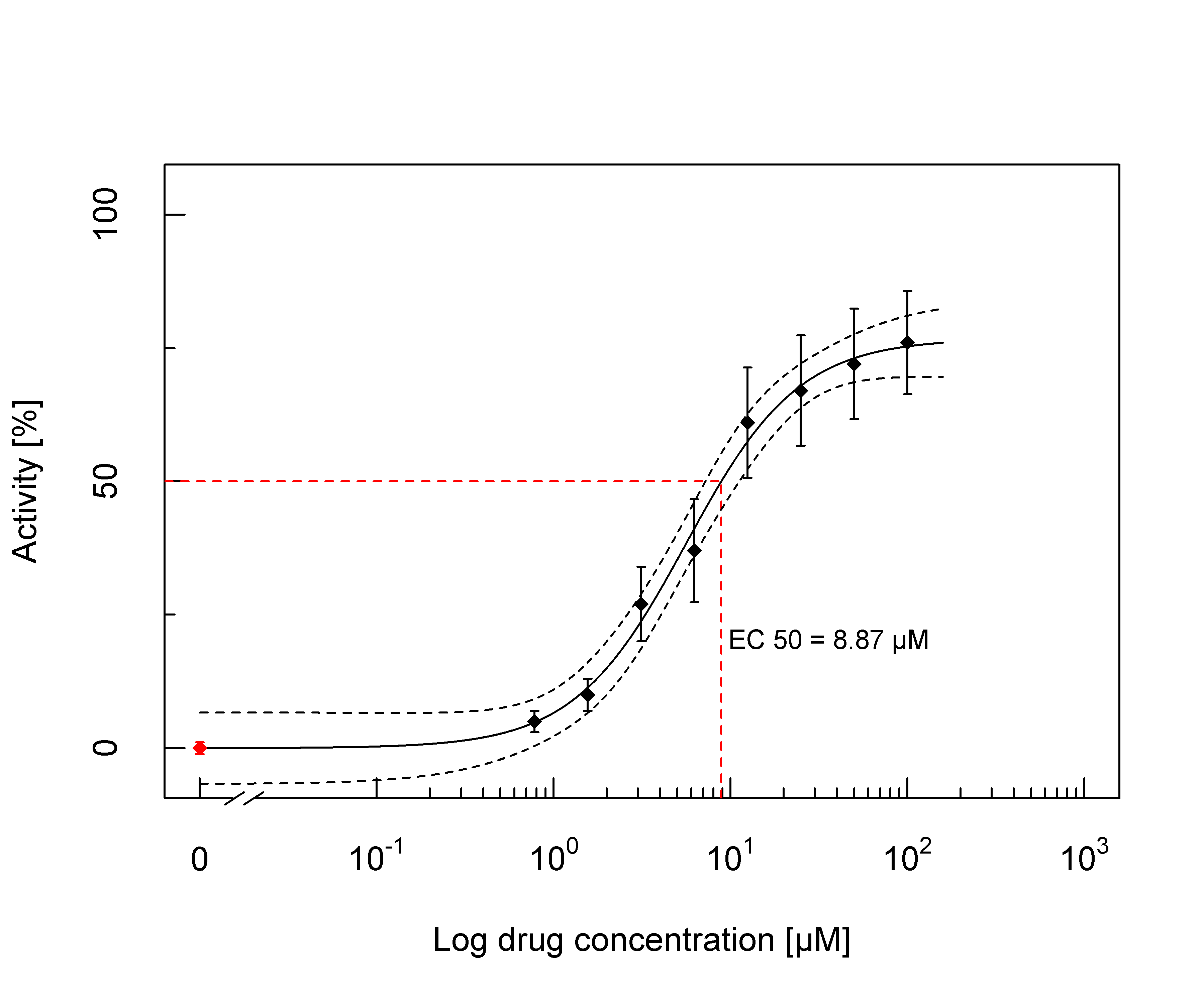


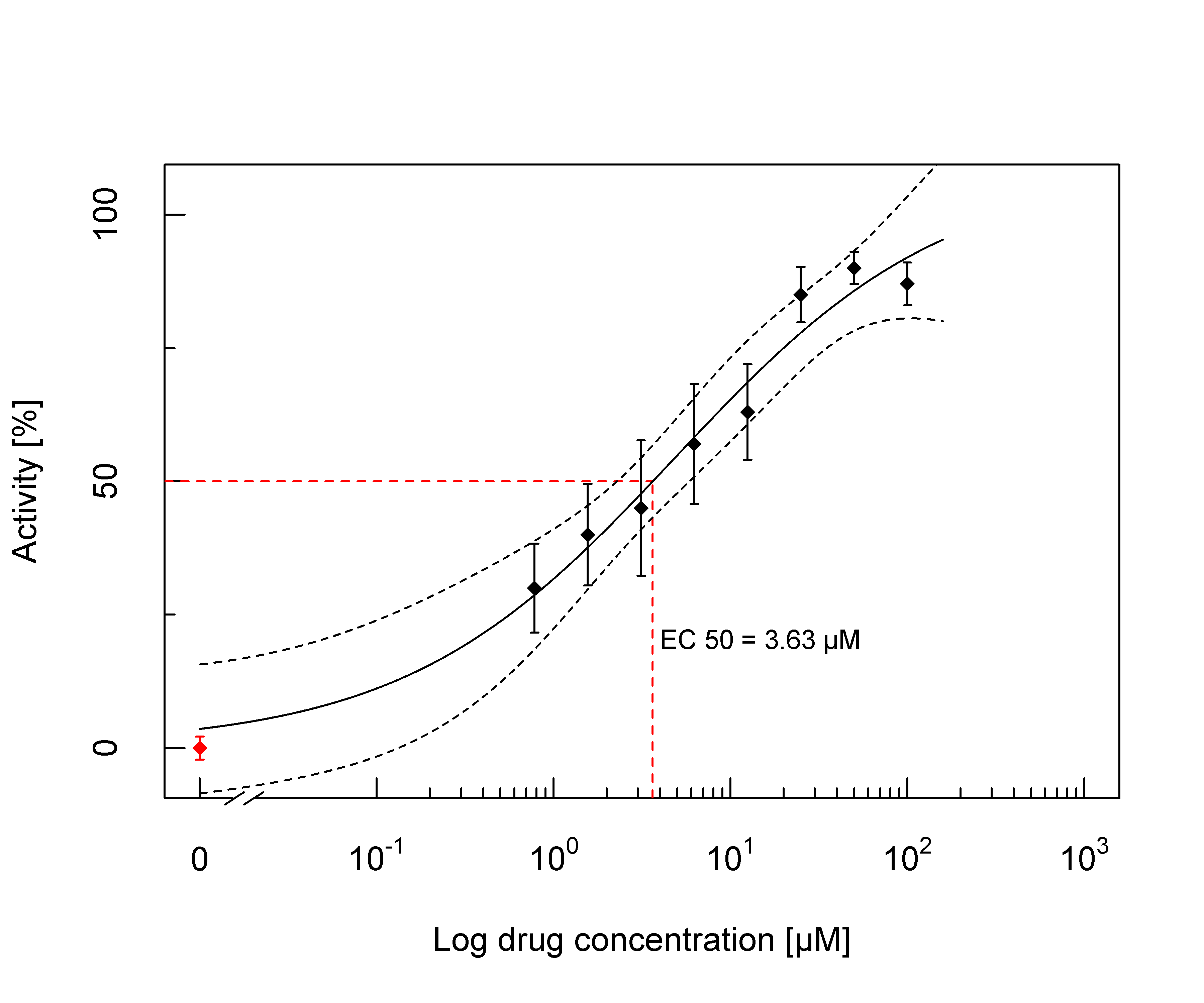

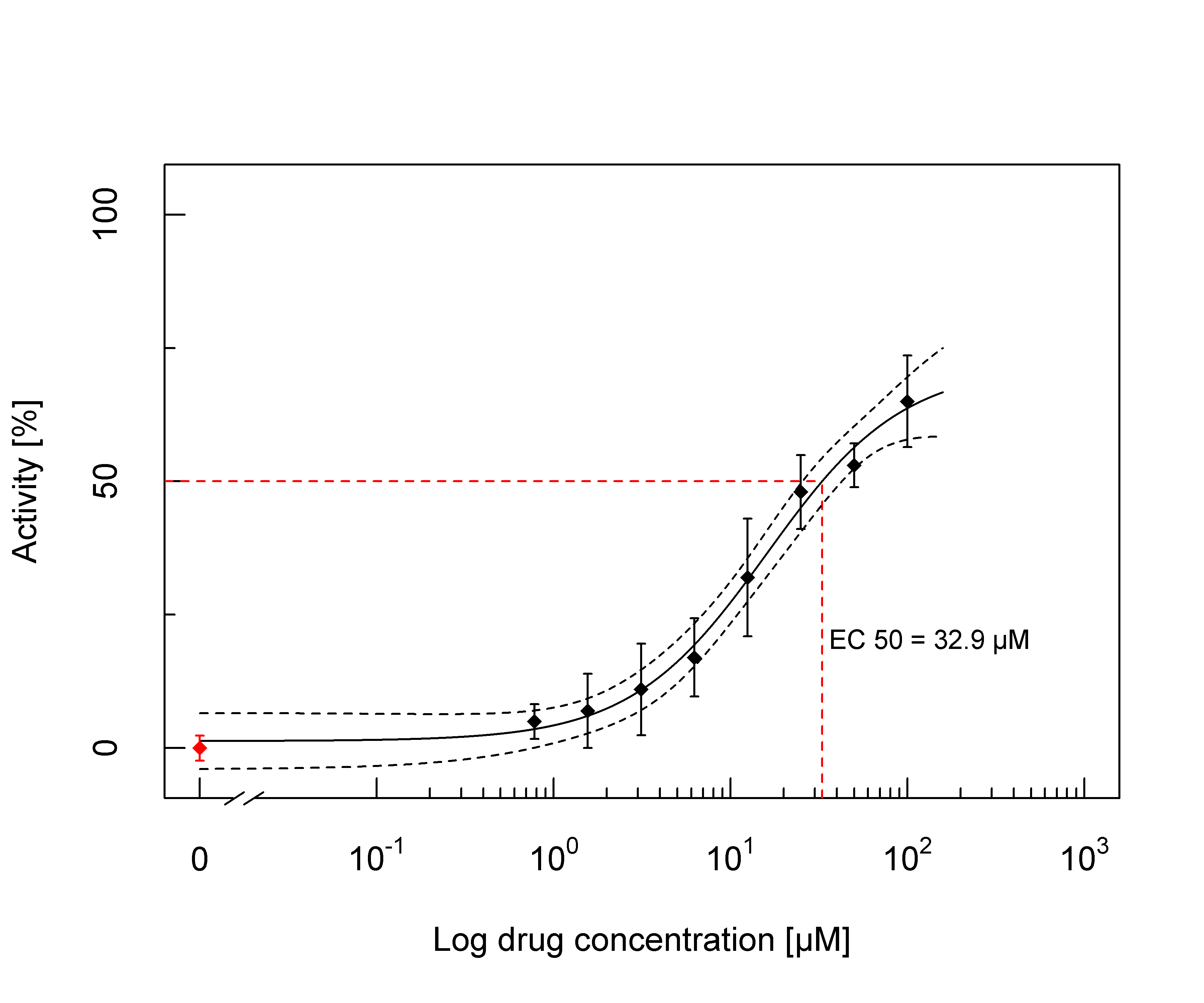


**Figure S10E:** Flubendazole.


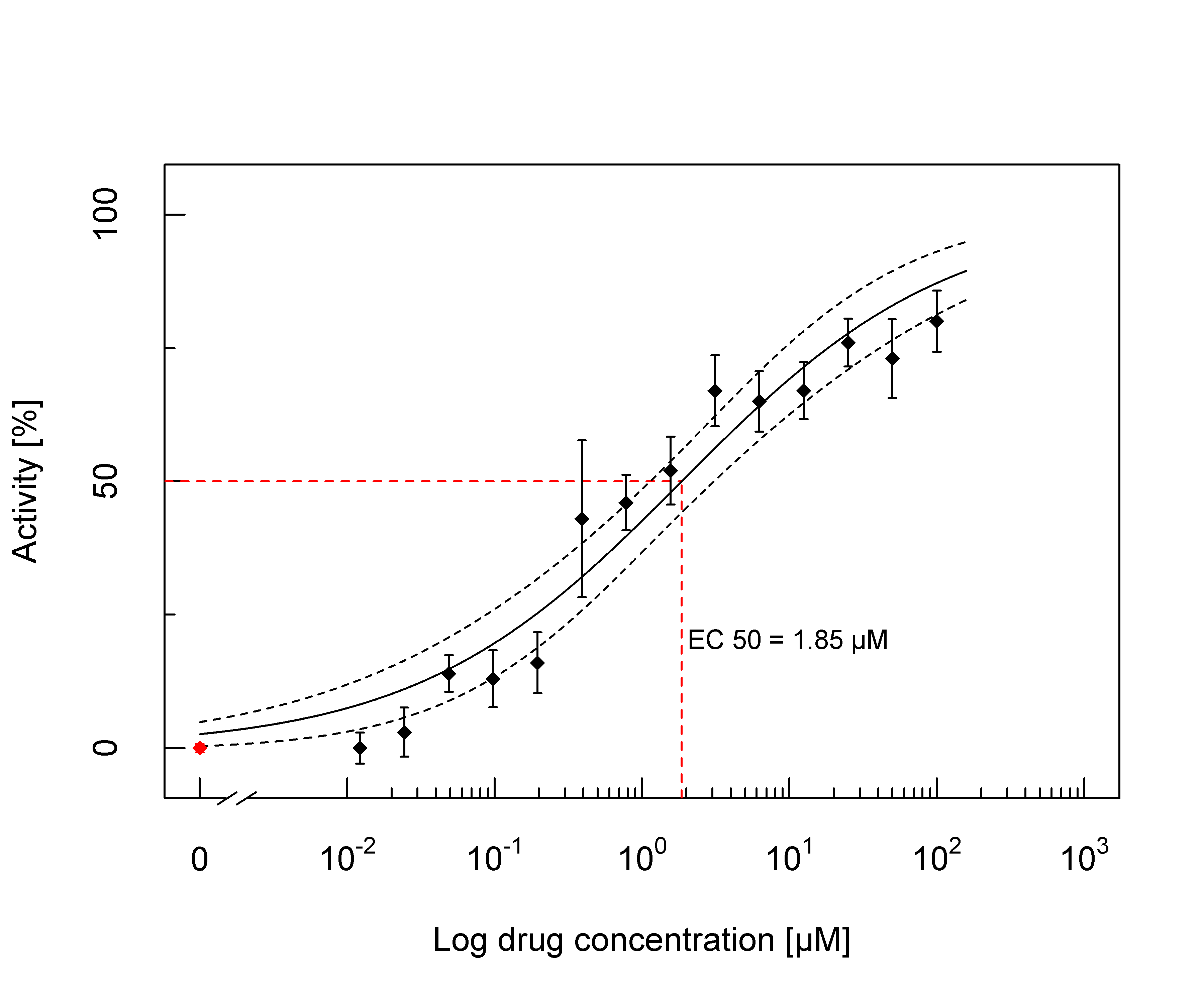


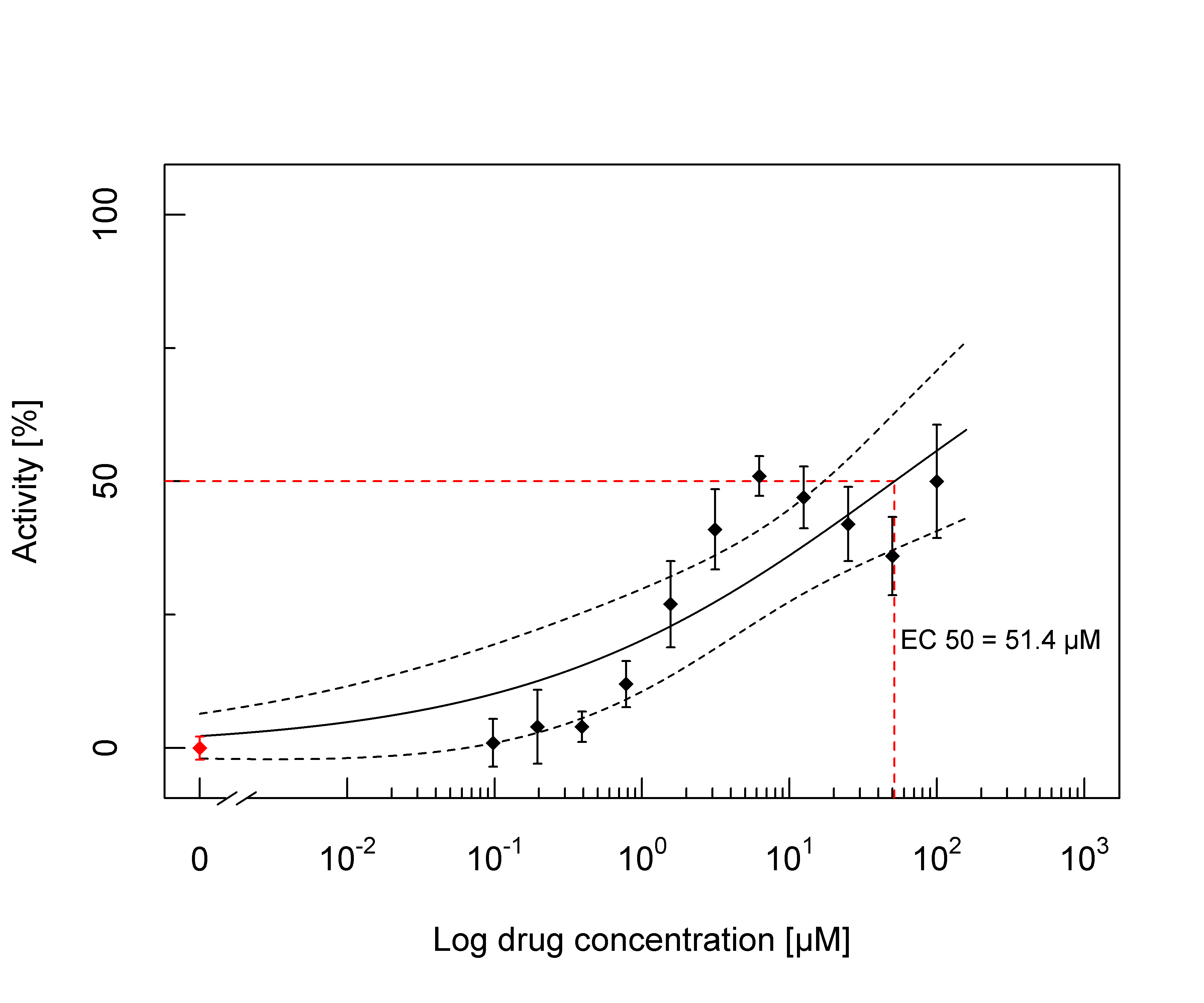

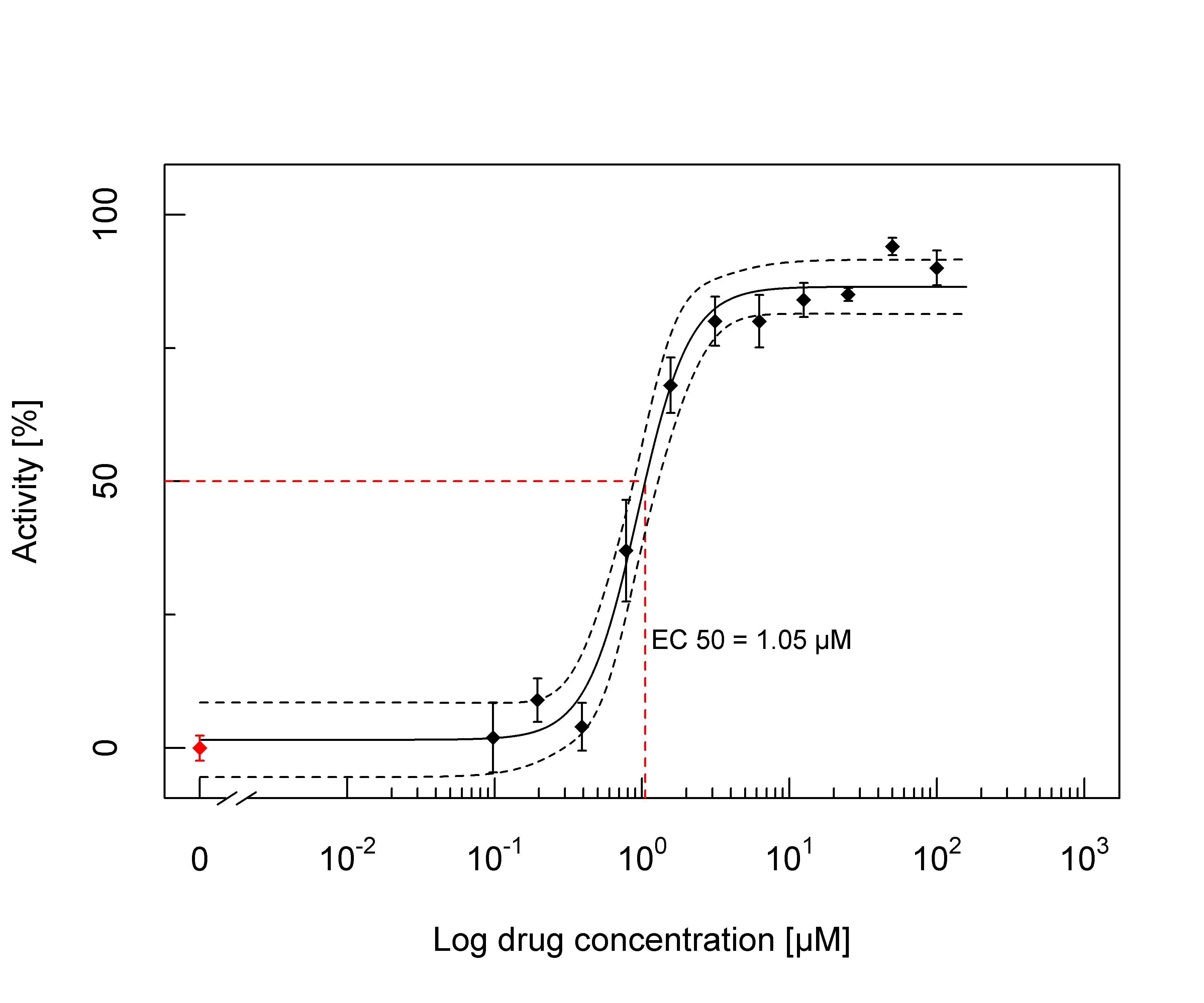


**Figure S10F:** Monepantel. Concentration-response curve not shown for

*H. polygyrus* unembryonated since EC_50_ was above 100 µM.


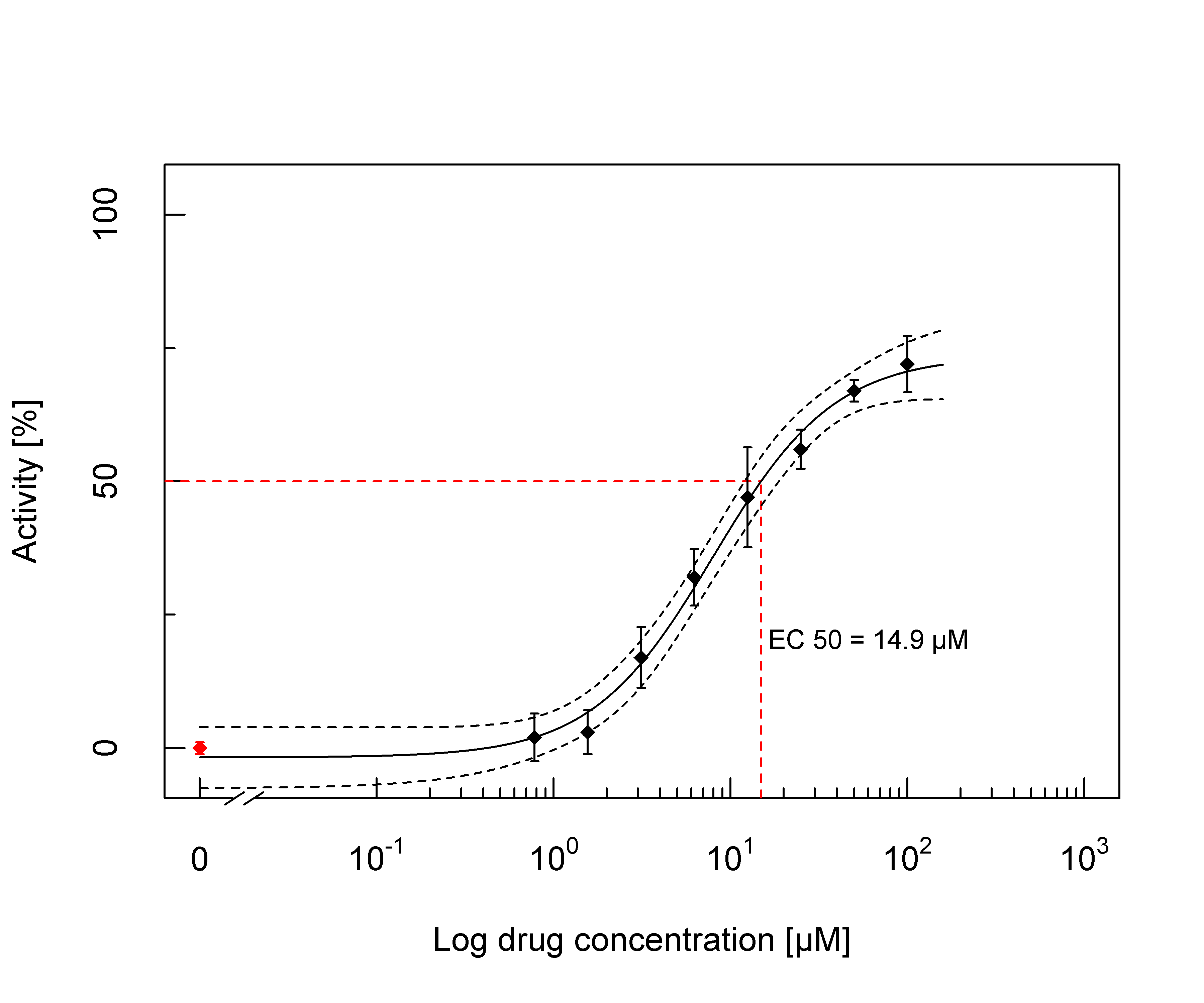

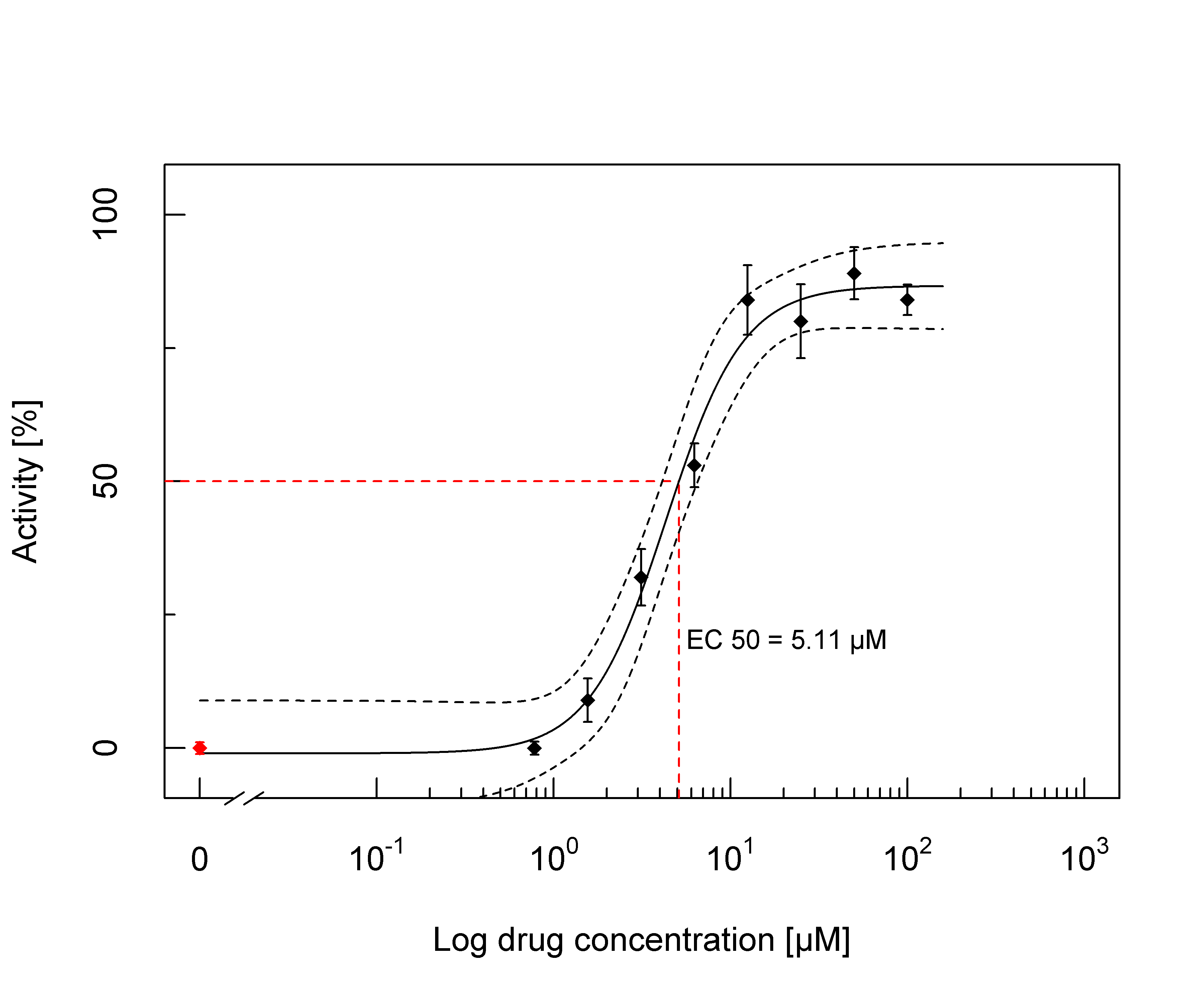


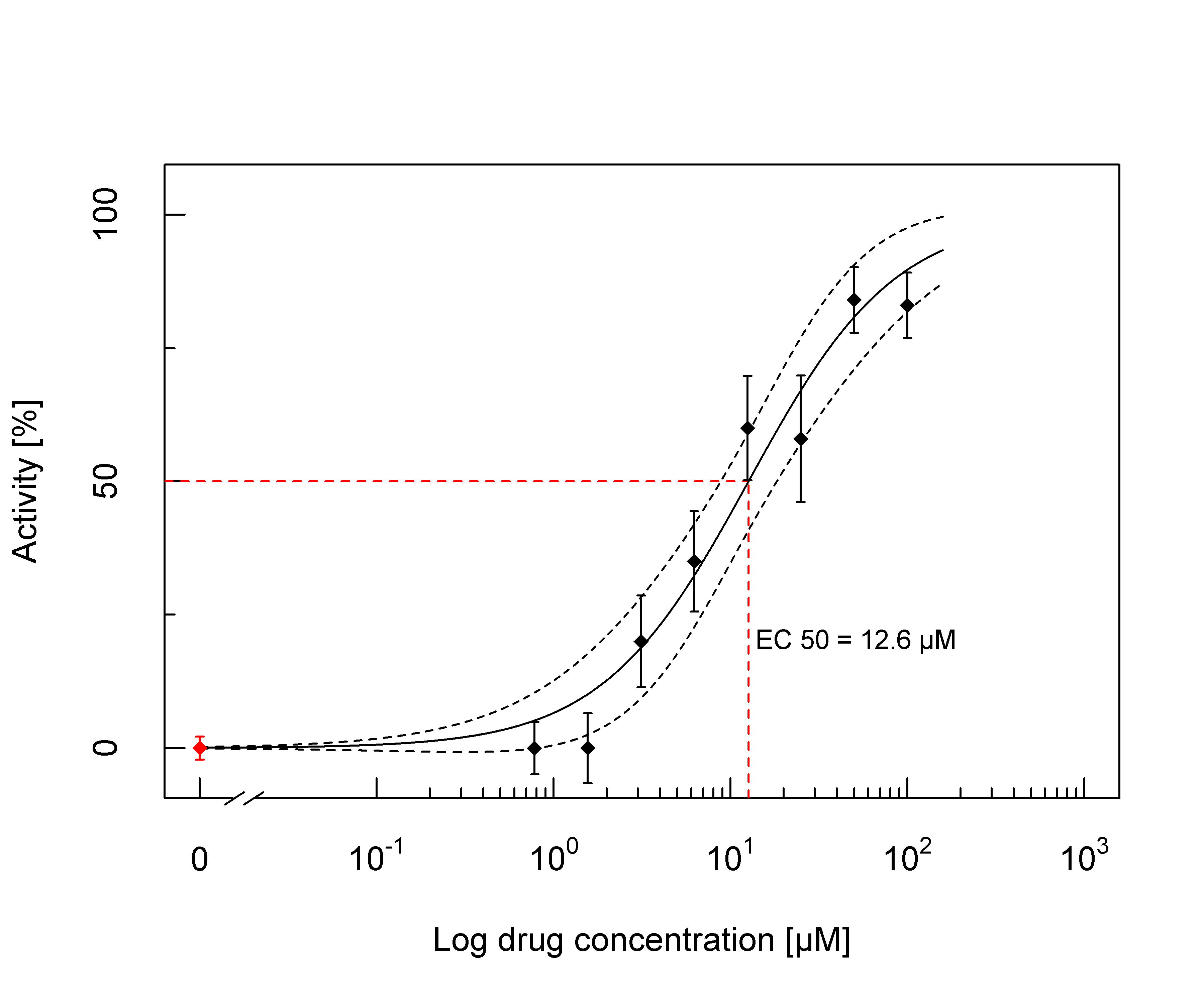

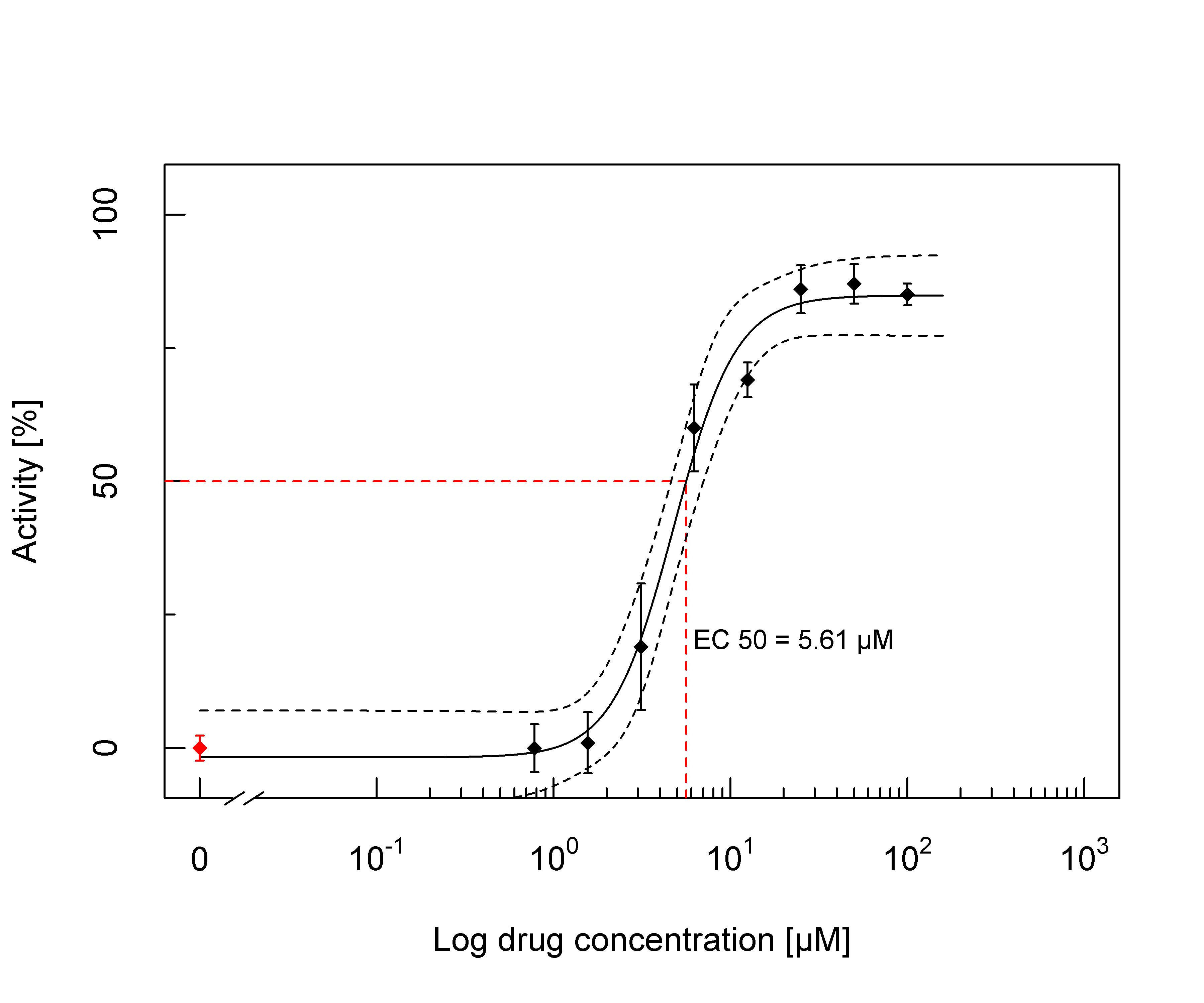


**Figure S10G:** Levamisole.


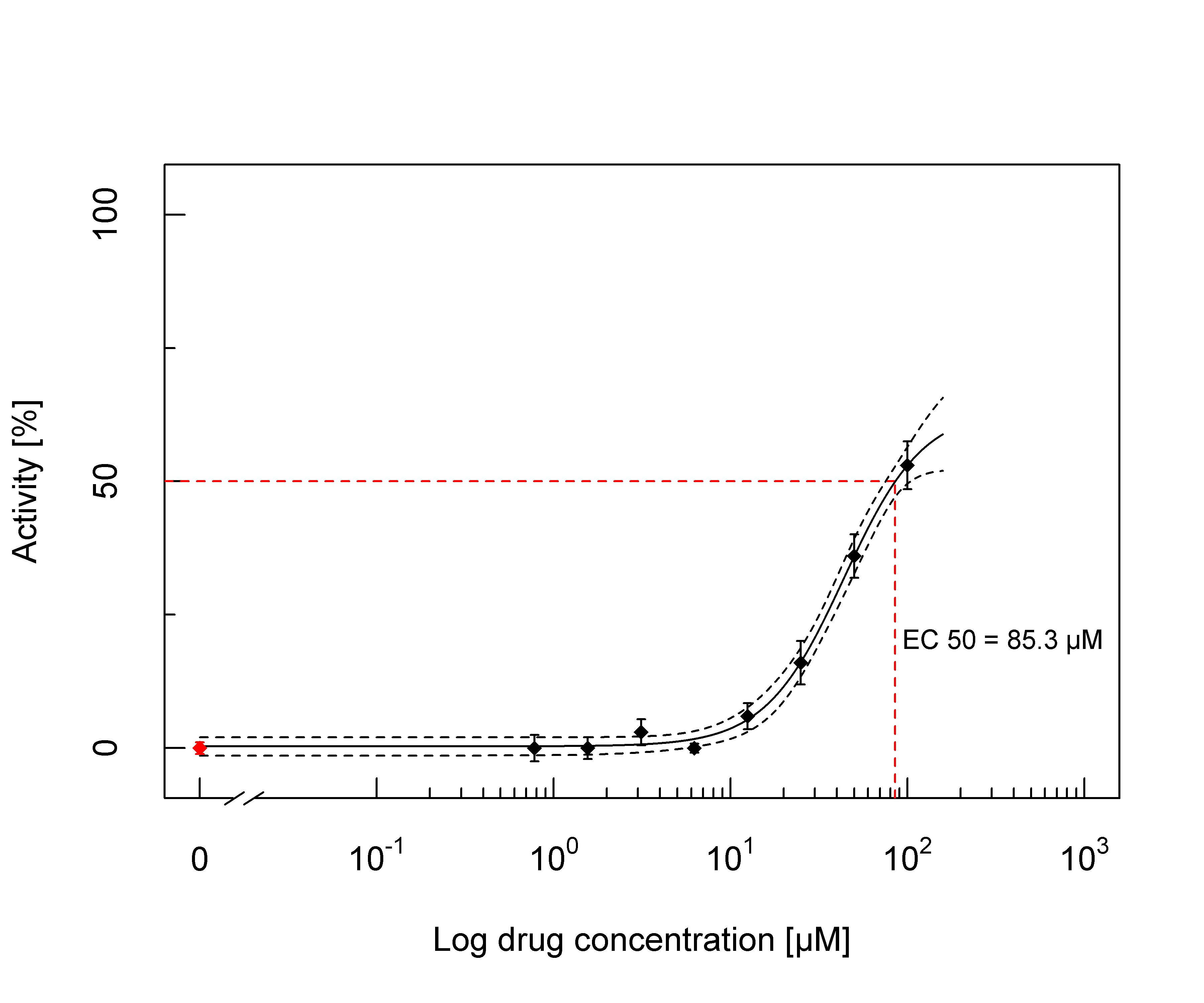

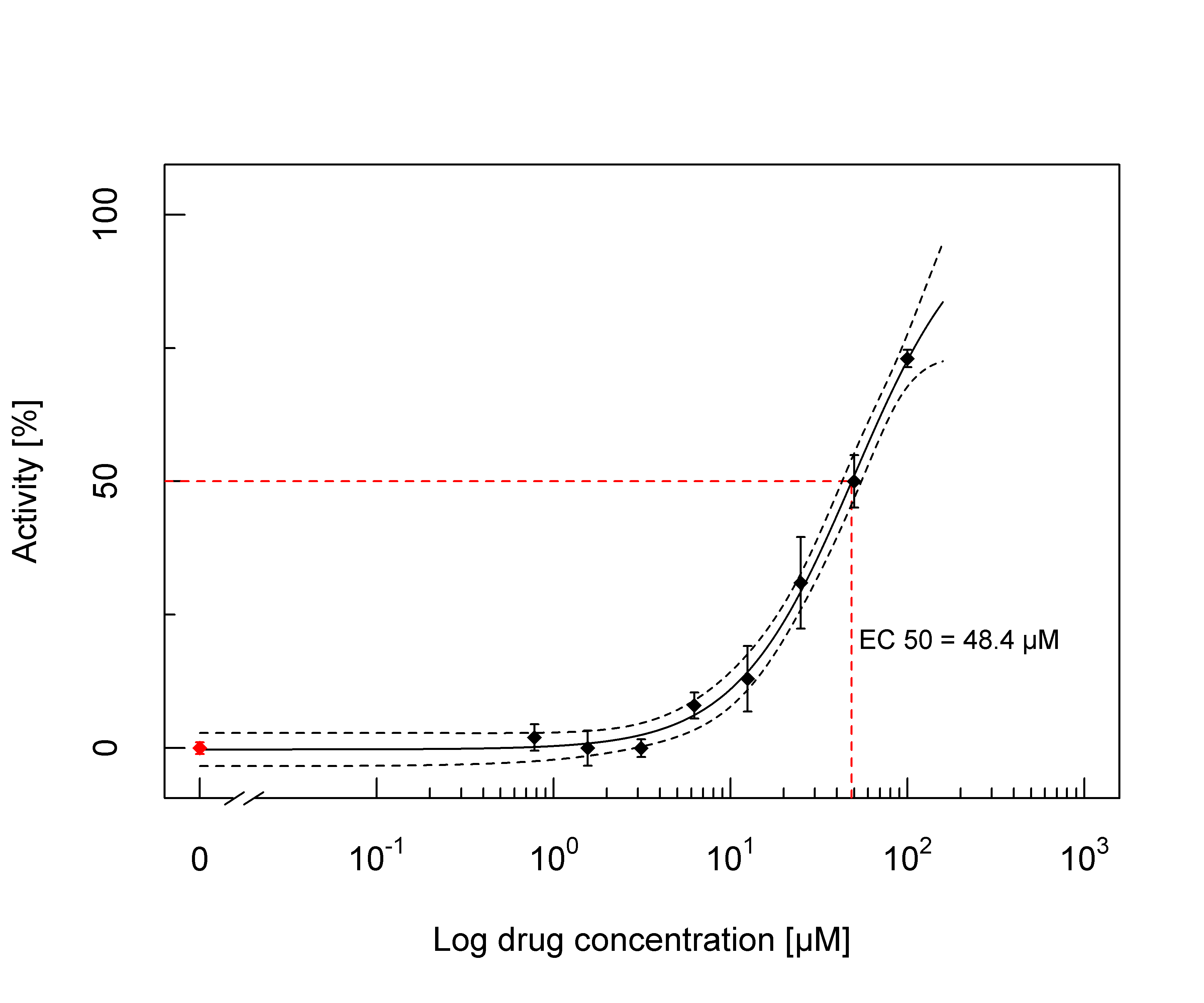


**
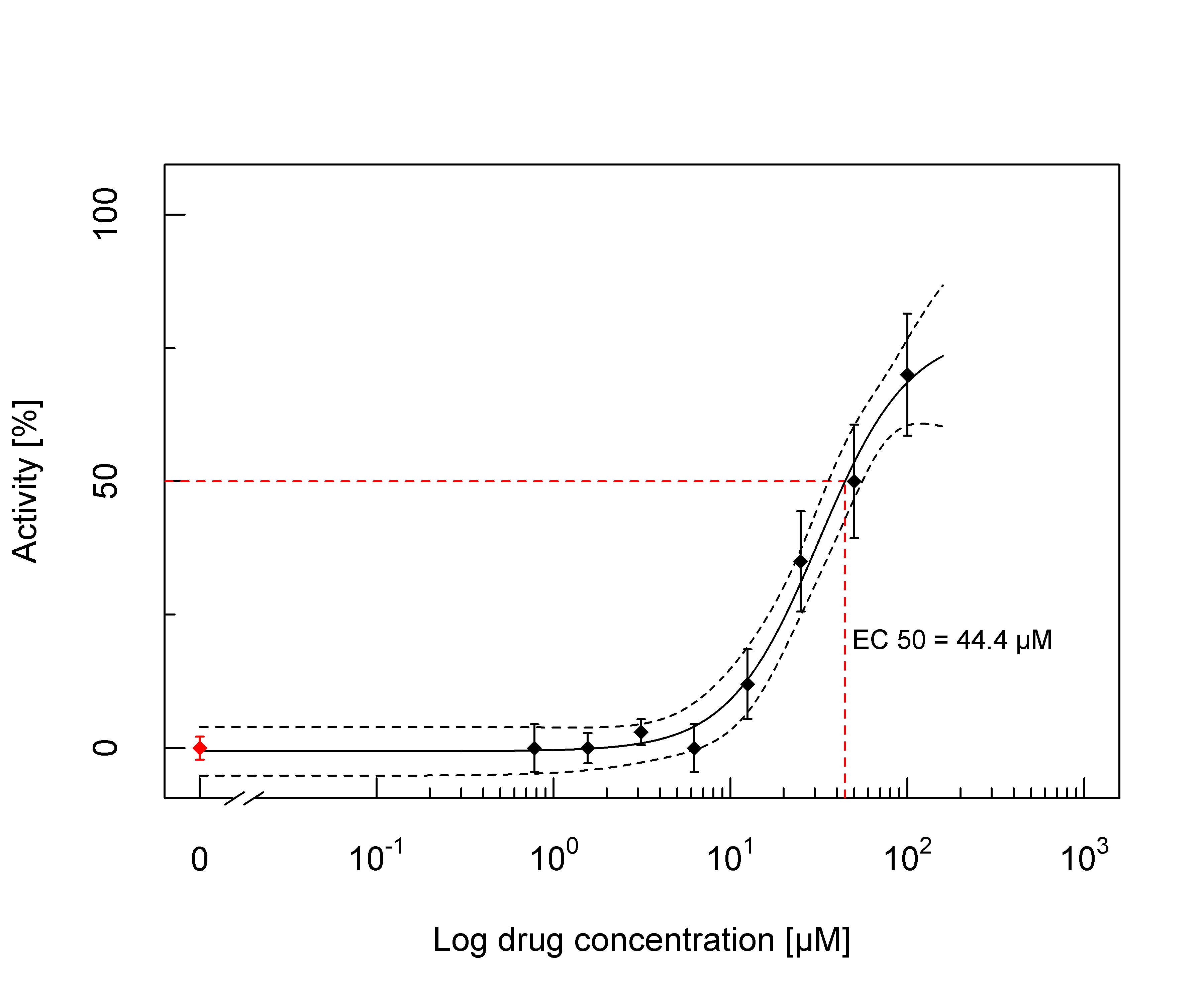
**

**Figure S10H:** Tribendimidine. Concentration-response curve not shown for

*A. ceylanicum* since EC_50_ was above 100 µM.

**Table S1:** Compound concentration range for *in vitro* EC_50_ determination.

| **Name** | **Upper** | **Lower** | **Dilution** |
| --- | --- | --- | --- |
|  | **Concentration [µM]** | |  |
| Flubendazole | 100 | 0.78 | 1:2 |
| Levamisole | 100 | 0.78 | 1:2 |
| Tribendimidine | 100 | 0.78 | 1:2 |
| Mebendazole | 100 | 0.1 | 1:2 |
| Monepantel | 100 | 0.1 | 1:2 |
| Albendazole | 100 | 0.001 | 1:2 |
| Oxibendazole | 100 | 0.001 | 1:2 |
| Thiabendazole | 100 | 0.001 | 1:2 |
